# Construction and validation of a novel prognostic model for lung squamous cell cancer based on N6-methyladenosine-related genes

**DOI:** 10.1186/s12957-022-02509-1

**Published:** 2022-02-27

**Authors:** Erna Jia, Na Ren, Bo Guo, Zhi Cui, Boyin Zhang, Jinru Xue

**Affiliations:** 1 Department of Gastroenterology, The Third Hospital of Jilin University, Changchun, China; 2 Department of Thoracic Surgery, The Third Hospital of Jilin University, No. 126, Xiantai Street, Changchun, Jilin, China; 3 Department of Orthopedic Surgery, The Third Hospital of Jilin University, Changchun, China

**Keywords:** m6A-related gene, Lung cancer, Prognosis, TCGA, GEO

## Abstract

**Background:**

N6-methyladenosine (m6A) is the most prevalent modification in mRNA in biological processes and associated with various malignant tumor initiation and progression. The present study aimed to construct a prognostic risk model based on m6A-related genes (the downstream genes influenced by m6A modulators) for LUSC.

**Methods:**

Based on TCGA, we stratified LUSC patients with and without genetic alteration of m6A modulators into altered and unaltered groups. Using univariate Cox and Lasso regression analyses, we identified prognostic m6A-related genes to construct a prognostic risk model. We then applied a multivariate Cox proportional regression model and the survival analysis to evaluate the risk model. Moreover, we performed the Receiver operating characteristic curve to assess the efficiency of the prognostic model based on TCGA and GSE43131. We analyzed the characteristics of tumor-associated immune cell infiltration in LUSC through the CIBERSORT method.

**Results:**

Three m6A-related genes (*FAM71F1*, *MT1E*, and *MYEOV*) were identified as prognostic genes for LUSC. A novel prognostic risk model based on the three m6A-related genes was constructed. The multivariate Cox analysis showed that the prognostic risk model was an independent risk factor (HR = 2.44, 95% CI = 1.21~3.56, *p* = 0.029). Patients with a high-risk group had worse overall survival both in TCGA (*p* = 0.018) and GSE43131 (*p* = 0.00017). The 1, 2, and 3-year AUC value in TCGA was 0.662, 0.662, and 0.655, respectively; The 1, 2, and 3-year AUC value in GSE43131 was 0.724, 0.724, and 0.722, respectively. The proportion of infiltrated neutrophils in the high-risk group was higher than that in the low-risk group (*p* = 0.028), whereas that of resting NK cells (*p* = 0.002) was lower.

**Conclusion:**

A novel prognostic risk model based on three m6A-related genes for LUSC was generated in this study.

**Supplementary Information:**

The online version contains supplementary material available at 10.1186/s12957-022-02509-1.

## Introduction

Non-small cell lung cancer (NSCLC) is the most common subtype (85%) of lung cancer, which is the most frequent and leading cause of cancer-related death worldwide [1]. Lung squamous cell cancer (LUSC), one of the predominant histological subtypes of NSCLC, accounts for 30% of NSCLC cases and 23% of all lung cancers in the United States of America [2]. Despite the demonstrated survival benefits of neo-adjuvant therapy, the 5-year survival rate of patients with LUSC remains dismal because the majority of such patients are diagnosed at an advanced stage with metastasis [3–5].. Although the clinical tumor-node-metastasis (TNM) stage is associated with the prognosis of LUSC, it cannot be used as an indicator to precisely estimate the overall survival [6, 7]. Developments in next-generation sequencing technology facilitate epigenetic markers to guide the evaluation of prognosis of malignant tumors [8, 9]. Therefore, integrating epigenetic-related genetic biomarkers to existing prognostic indicators would be beneficial to improving the accuracy of estimating the overall survival of patients with LUSC.

N6-Methyladenosine (m6A) is the most abundant internal modification on mRNAs in eukaryotic cells [10]. It participates in almost all steps of mRNA metabolism, such as translation, spicing, folding, degradation, and export. M6A modification is a complicated dynamic and reversible process, which has emerged as a vital regulator of gene expression influencing biological processes and pathological functions [11, 12]. M6A can be catalyzed by the methyltransferases (also termed writers: METTL3, METTL14, WTAP, etc.), recruited by m6A-binding proteins (also termed readers: FTO and ALKBH5 ), and removed by demethylases (also termed erasers: YTHDC1/2, YTHDF1/2/3, and IGF2BP1/2/3, etc.) [13]. Emerging evidence has demonstrated that dysregulation of M6A modulators, such as YTHDF1, METTL3, and FTO, plays an important role in the regulation of the occurrence, progression, and immune microenvironment of malignant tumors [14–19]. Although the underlying mechanisms of dysregulation m6A modulators in malignant tumors remain elusive, the relationship between m6A modulators and malignant tumors has been verified in many researches. However, few studies have investigated the association of malignant tumors with downstream genes influenced by m6A modulators, and the role of such genes in prognostic evaluation of LUSC is still unclear.

The present study aims to investigate the impact of genetic alteration of m6A modulators on the overall survival of LUSC patients, as well as the effect of downstream genes influenced by m6A modulators on prognosis of LUSC, using data from The Cancer Genome Atlas (TCGA) and Gene Expression Omnibus (GEO) databases, and to construct a prognostic risk model for LUSC based on differently expressed m6A-related genes.

## Materials and methods

### Data profiles

**Fig. 1:**
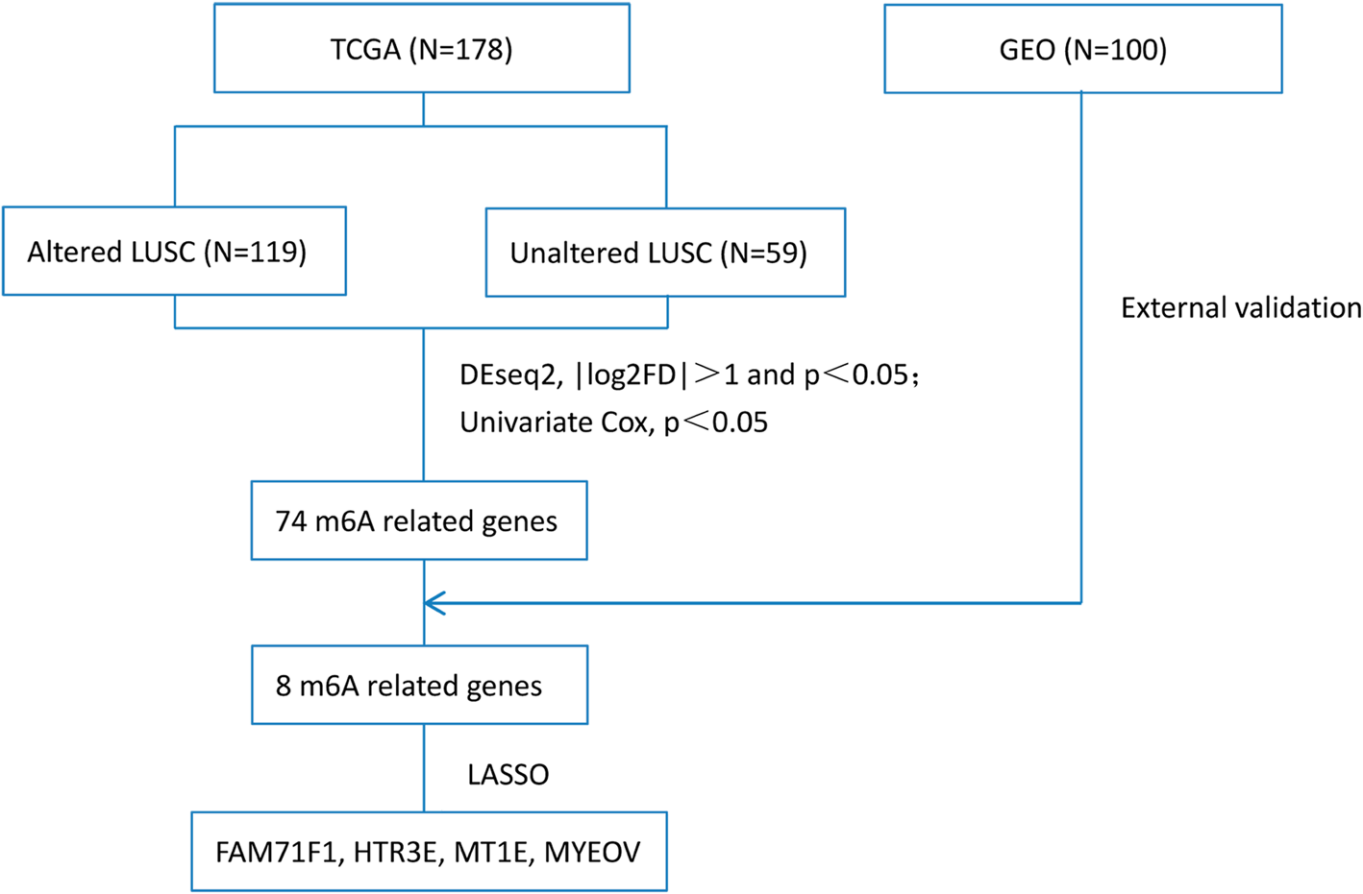
Flow chart of the study

We downloaded raw RNA sequencing data (fragments per kilobase of exon model per million reads mapped [FPKM] and count data), copy number variation (CNV) and mutation data, and corresponding information on clinicopathological features (including sex, age, race, smoking history, cancer status, pathologic stage, T stage, N stage, M stage, survival time, and survival status) from TCGA (https://www.cancer.gov, LUSC project) and a GEO external validation dataset (https://www.ncbi.nlm.nih.gov/, GSE43131) on 5 Jan 2021. Finally, we obtained 178 and 100 LUSC samples, respectively. The study design is outlined in Fig. 1.

### Selection of m6A modulators and analysis of clinicopathological features

We selected 19 m6A modulators from published reports (i.e., METTL3, METTL14, METTL16, WTAP, RBM15, RBM15B, CBLL1, VIRMA, ZC3H13, FTO, ALKBH5, YTHDC1, YTHDC2, YTHDF1, YTHDF2, YTHDF3, IGF2BP1, IGF2BP2, and IGF2BP), which were included in our analysis. LUSC patients with and without genetic alterations (mutation and/or CNV) of m6A modulators from TCGA were stratified into the altered and unaltered groups, respectively. We analyzed the difference of the overall survival between the two groups through the log-rank test and survival curves were generated using the Kaplan–Meier method. Differences in clinicopathological features between the two groups were determined using the chi-squared or Fisher’s exact test. A *p-*value (two-sided) < 0.05 denoted statistically significant difference.

### Construction of a prognostic risk score model and the ROC curves for LUSC prognosis

We normalized raw RNA sequencing count data obtained from TCGA LUSC and searched for the downstream genes influenced by m6A modulators (defined m6A-related genes) between altered and unaltered groups using the R “Deseq2” package; *p* < 0.05 and |log_2_ (false discovery rate)| > 1 denoted statistical significance. Univariate Cox regression analysis was conducted to identify prognostic m6A-related genes in TCGA LUSC; *p*<0.1 denoted statistical significance. We validated the prognostic m6A-related genes in GSE43131; genes with *p*<0.1 were considered candidate prognostic m6A-related genes. Furthermore, least absolute shrinkage and selection operator (LASSO) regression analysis was performed to select target prognostic m6A-related genes for the construction of a prognostic risk model for LUSC. Furthermore, we verified the selected target prognostic m6A-related genes which also differently expressed between LUSC and adjacent normal tissues in TCGA dataset using “limma” package by R software (adj. *p*. value < 0.05 and |log_2_ (fold change)| > 1 denoted statistical significance). The following calculation formula was used:

$$Risk\ score={\sum}_{ni}=\sum \left({\mathrm{coef}}_i+{\upchi}_i\right)$$

where *coef*_*i*_ is the LASSO Cox regression coefficient and *χ*_*i*_ is the expression value of each selected m6A-related gene. We used “glmnet” and “survival” packages by R software to calculate the coefficients of the m6A-related genes involved in the risk score. Finally, we calculated a risk score for each patient with LUSC both in TCGA and GSE43131.

To test the prognostic significance of the risk model, we divided LUSC patients from TCGA into high- and low-risk groups based on the median risk score; those with survival time < 180 days were excluded. Using the R “survival” package, we compared the overall survival between the two groups through the Kaplan–Meier plotter with log-rank *p-*value. We also produced receiver operating characteristic (ROC) curves with area under the curve (AUC) values for 1-, 2-, and 3-year overall survival to evaluate the prognostic performance of the risk model for LUSC. Moreover, we validated the prognostic value of the risk model for LUSC in the GSE43131 using similar statistical analyses, in which we also excluded LUSC patients with survival time < 180 days (6 months).

To further address the prognosis association of the risk model, we conducted univariate and multivariate Cox proportional hazard analyses. These analyses were performed to verify independent risk factors for LUSC in TCGA, with covariates including the risk score, sex, age, race, smoking history, cancer status, pathologic stage, T stage, N stage, and M stage.

### Estimation of differences in immune cell infiltration between the high- and low-risk score groups

The tumor immune microenvironment has been associated with overall survival in almost all types of cancer, with different proportions of immune cell infiltration indicating either poor or good prognostic performance. Herein, we converted the FPKM format of RNA sequencing data of LUSC from TCGA into the transcripts per million (TPM) format, uploaded the TPM data to the CIBERSORT (Cell-type Identification By Estimating Relative Subsets Of known RNA Transcripts) online tool, and investigated differences in the relative infiltration of 22 types of immune cells between the high- and low-risk groups. Subsequently, we inferred the relationships between the overall survival of LUSC patients and the immune cell infiltration. CIBERSORT is a novel classic bulk RNA deconvolution tool that is based on linear support vector regression to statistically estimate the relative infiltration of immune cell subsets from the expression profiles of bulk tumors [20]. This approach provides a gene signature matrix of 22 leukocyte subsets (LM22), and a total of 547 gene signature expression values are set as references.

### Function enrichment analyses

To investigate the potential biological mechanisms of m6A-related genes involved in the occurrence, progression, and metastasis of LUSC, we conducted Gene Ontology (GO) enrichment and Kyoto Encyclopedia of Genes and Genomes (KEGG) pathway analyses using the R “clusterProfiler” package.

### Statistical analyses

We used the R software (version 3.5.3 and 4.0.3; R Foundation, Vienna, Austria) to perform all statistical calculations and produce the corresponding figures.

## Results

### Alteration features of m6A modulators in LUSC from TCGA dataset

**Fig. 2:**
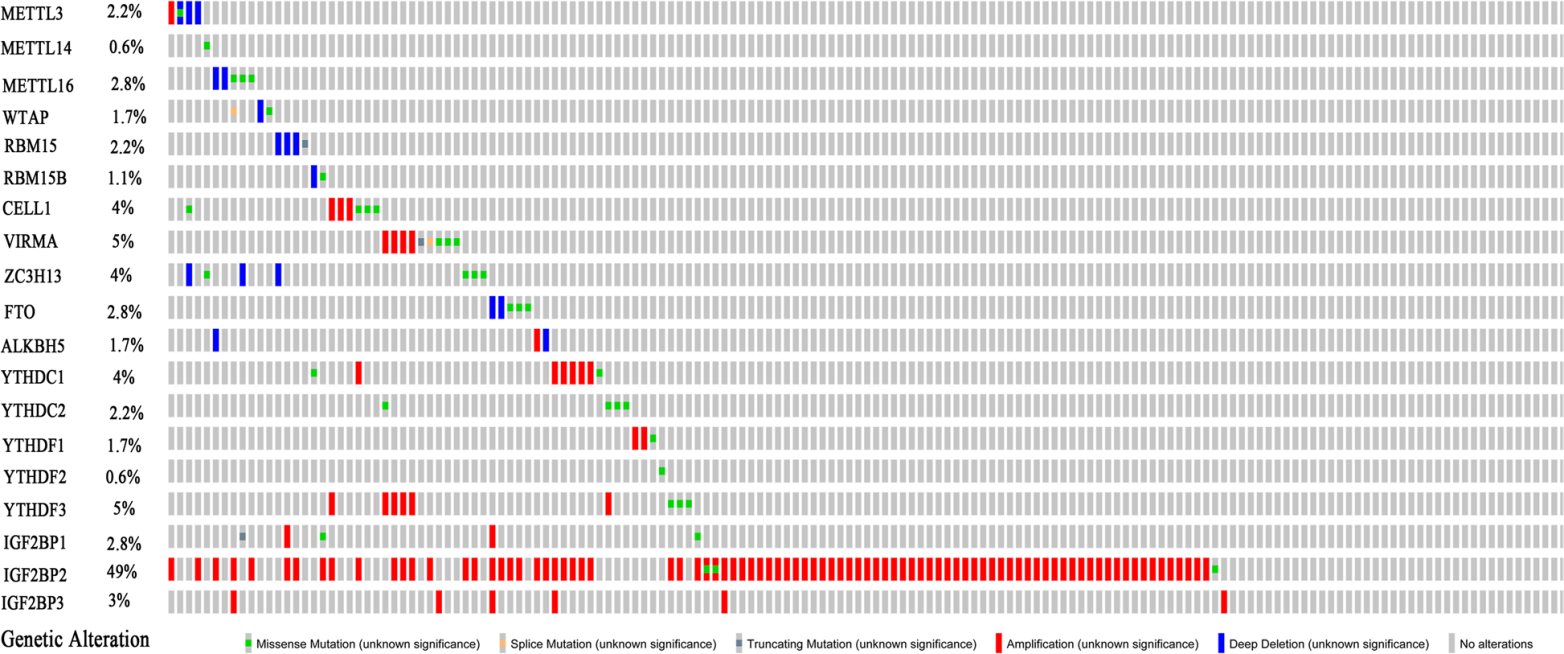
Genetic alteration signatures of m6A modification genes in samples of lung squamous cell cancer from TCGA dataset. TCGA, The Cancer Genome Atlas

**Fig. 3:**
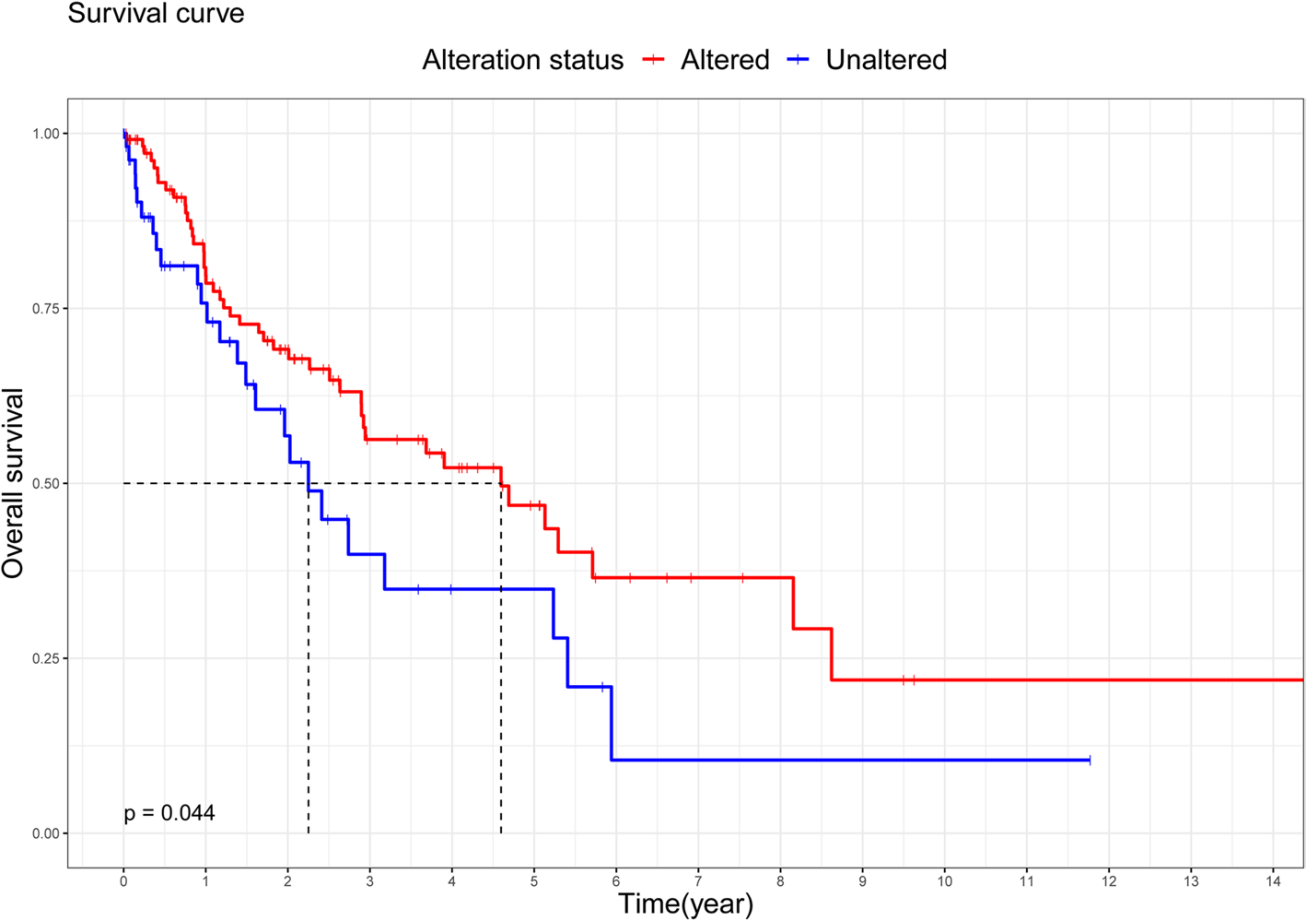
Kaplan–Meier overall survival analysis of 178 patients with lung squamous cell cancer from TCGA dataset. TCGA, The Cancer Genome Atlas

We investigated 119 and 59 samples of LUSC from TCGA with and without alterations of m6A modulators, respectively (altered and unaltered groups, respectively). Detailed information on these alterations was shown in Table 1 and Fig. 2. IGF2BP2 was the most frequently altered m6A modulator (50.56%), followed by YTHDF3 (5.06%), VIRMA (5.06%), YTHDC1 (4.49%), ZC3H13 (3.93%), and CBLL1 (3.93%). The most frequent form of alteration was amplification (Table 1 and Fig. 2). Interestingly, a tendency towards better overall survival was found in the altered group compared with the unaltered group (*p* = 0.044; Fig. 3). We also compared the two groups in terms of clinicopathological features (including sex, age, race, smoking history, cancer status, pathologic stage, T stage, N stage, and M stage); the results did not reveal statistically significant differences (Table 2).

**Table 1:** Genetic alteration signatures of m6A modification genes in samples of lung squamous cell cancer from TCGA (*n* = 178)

			Genetic alteration					
			Mutation			CNV		
		No alterations	Missense mutation	Splice	Truncating mutation	Amplification	Deep deletion	Altered/profiled
**Writer**	METTL3	173	1	0	0	1	3	2.81%
	METTL14	177	1	0	0	0	0	0.56%
	METTL16	173	3	0	0	0	2	2.81%
	WTAP	175	1	1	0	0	1	1.69%
	RBM15	174	0	0	1	0	3	2.25%
	RBM15B	176	1	0	0	0	1	1.12%
	CBLL1	171	4	0	0	3	0	3.93%
	VIRMA	169	3	1	1	4	0	5.06%
	ZC3H13	171	4	0	0	0	3	3.93%
**Eraser**	FTO	173	3	0	0	0	2	2.81%
	ALKBH5	175	0	0	0	1	2	1.69%
**Reader**	YTHDC1	170	2	0	0	6	0	4.49%
	YTHDC2	174	4	0	0	0	0	2.25%
	YTHDF1	175	1	0	0	2	0	1.69%
	YTHDF2	177	1	0	0	0	0	0.56%
	YTHDF3	169	3	0	0	6	0	5.06%
	IGF2BP1	173	2	0	1	2	0	2.81%
	IGF2BP2	88	3	0	0	87	0	50.56%
	IGF2BP3	172	0	0	0	6	0	3.37%

**Table 2:** Clinicopathological features of altered and unaltered patients with LUSC from TCGA

		Unaltered(***n*** = 59)	Altered(***n*** = 119)	***p***-value
Sex				0.278
	Female	19	28	
	Male	40	91	
Age				0.386
	< 65	15	40	
	≥ 65	42	78	
	NA	2	1	
Race				0.769
	White	41	72	
	Non-white	4	10	
	NA	14	37	
Smoking_history				1.000
	Yes	57	116	
	No	2	3	
Cancer_status				0.658
	With cancer	10	18	
	Cancer free	49	101	
Pathologic_stage				0.978
	I	34	65	
	II	12	27	
	III	12	23	
	IV	1	2	
	NA	0	2	
T stage				0.539
	T1	16	21	
	T2	35	80	
	T3	4	10	
	T4	4	8	
N stage				0.521
	N0	40	76	
	N1	10	29	
	N2	6	11	
	N3	2	3	
	NX	1	0	
M stage				0.172
	M0	53	112	
	M1	1	2	
	MX	4	2	
	NA	1	3	

Altered, LUSC patients with mutation and/or copy number variation; Unaltered, LUSC patients without mutation and/or copy number variation; *LUSC* lung squamous cell cancer, *TCGA* The Cancer Genome Atlas

### Identification of seven prognostic m6A-related genes and construction of a risk score model

**Fig. 4:**
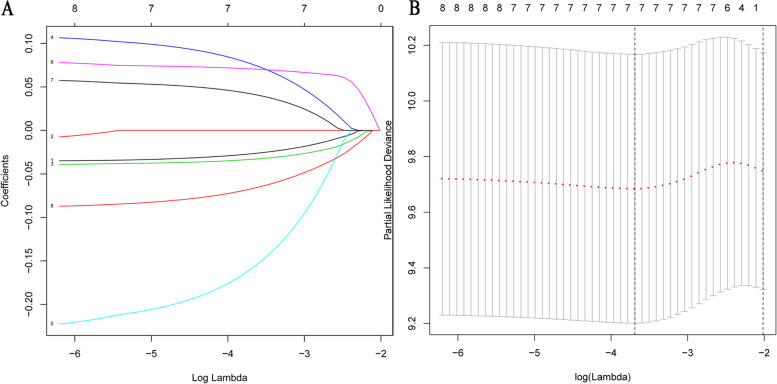
Distribution of Lasso coefficients for eight m6A-related genes. **A** Lasso coefficient spectrum of eight m6A-related genes in LUSC. **B** Cross-validation for tuning parameter selection in the proportional hazards model. Lasso, least absolute shrinkage and selection operator

We screened 872 m6A-related genes (373 down-regulated, 499 up-regulated) by comparing the altered and unaltered groups of LUSC patients from TCGA (Supplementary Table 1). The univariate Cox regression analysis identified 74 m6A-related genes (*p*<0.1) associated with overall survival of LUSC patients (Supplementary Table 2). We validated eight of these 74 prognostic m6A-related genes in the external validation dataset GSE43131 for further analysis (*p* < 0.1, Table 3). To avoid overfitting, we conducted LASSO regression analysis to reduced dimension and selected seven genes as target prognostic m6A-related genes (*CNTNAP2*, *FAM71F1*, *GRM4*, *HTR3E*, *MT1E*, *MYEOV*, and *POU3F2*) based on the minimum criterion (Fig. 4 A and B). *MT1E* and *MYEOV* were identified as prognostic genes of risk in both TCGA and GSE43131 datasets (hazard ratio [21] >1; Table 3). In contrast, *FAM71F1* and *HTR3E* were identified as prognostic genes of protection in both datasets (HR < 1; Table 3). Notably, *CNTNAP2*, *GRM4*, and *POU3F2* exhibited opposite prognostic associations in the two datasets (Table 3). *FAM71F1*, *MT1E*, and *MYEOV* were also differently expressed between LUSC and normal tissues (Supplementary Table 3). Hence, we selected *FAM71F1*, *MT1E*, and *MYEOV* to generate a prognostic risk model: risk score = (− 0.03323723 × expression of *FAM71F1*) + (0.07068792 × expression of *MT1E*) + (0.04196110 × expression of *MYEOV*).

**Table 3:** Prognostic m6A-related genes for LUSC in TCGA and GSE43131

Gene symbol	TCGA-univariate Cox results			GEO-univariate Cox results	
	HR (95%CI)	*p*-value		HR (95%CI)	*p*-value
*CNTNAP2*	0.90(0.82~0.99)	0.029		24.28(1.83~32.18)	0.016
*EREG*	1.12(1.00~1.27)	0.064		0.14(0.02~0.84)	0.033
*FAM71F1*	0.90(0.81~1.01)	0.056		0.09(0.01~0.74)	0.025
*GRM4*	1.11(0.99~1.25)	0.066		0.13(0.01~1.27)	0.085
*HTR3E*	0.81(0.64~1.01)	0.057		0.28(0.07~1.17)	0.079
*MT1E*	1.23(1.03~1.46)	0.024		35.60(4.17~304.04)	0.001
*MYEOV*	1.11(0.99~1.25)	0.072		5.87(1.59~21.70)	0.012
*POU3F2*	0.90(0.80~1.00)	0.046		79.27(3.19~1969.46)	0.008

*CI* confidence interval, *GEO* Gene Expression Omnibus, *HR* hazard ratio, *LUSC* lung squamous cell cancer, *m6A* N6-methyladenosine, *TCGA* The Cancer Genome Atlas

### The ROC curves of the risk score model for LUSC prognosis

**Fig. 5:**
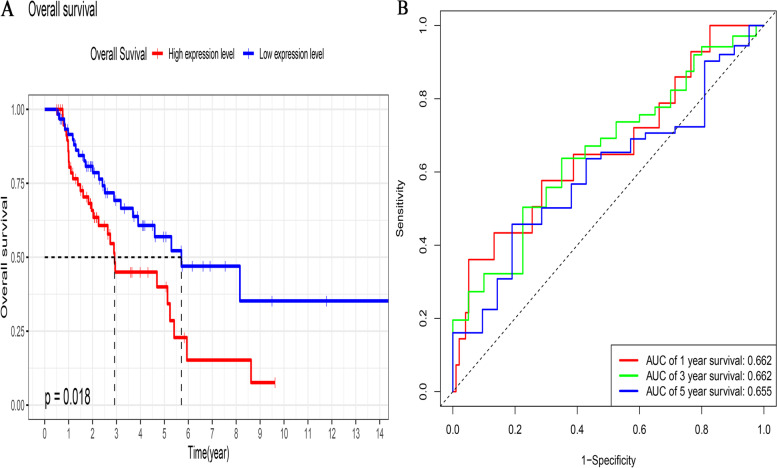
Overall survival between the high- and low-risk score subgroups according to the median risk score and ROC curves predicting survival in patients with lung squamous cell cancer from TCGA dataset. **A** Difference in overall survival between the two subgroups. **B** ROC curve predicting survival at 1, 2, and 3 years. ROC, receiver operating characteristic; TCGA, The Cancer Genome Atlas

**Fig. 6:**
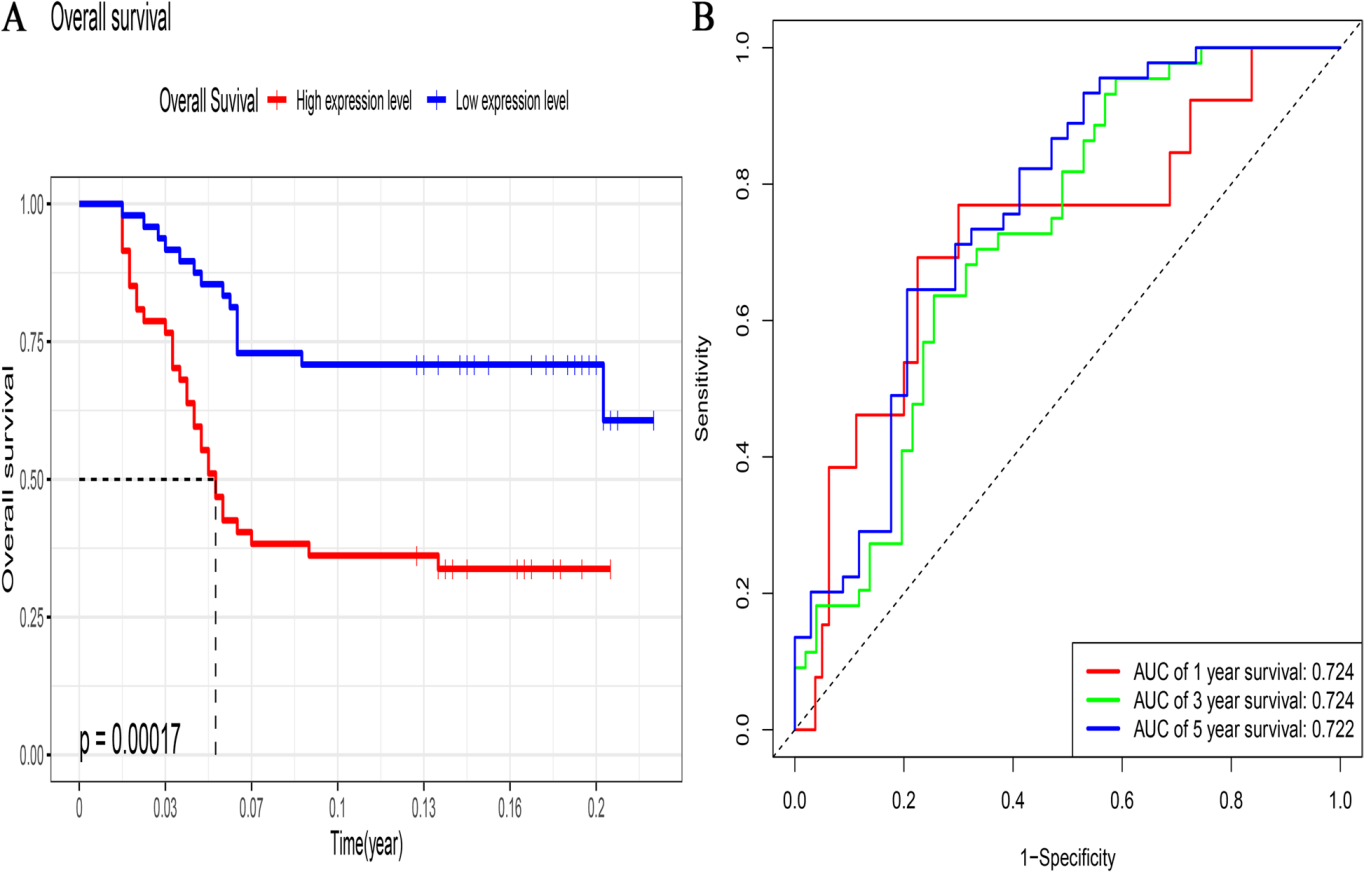
Overall survival between the high- and low-risk score subgroups according to the median risk score and ROC curves predicting survival in patients with lung squamous cell cancer from the GEO external validation dataset. **A** Difference in overall survival between the two subgroups. **B** ROC curve predicting survival at 1, 2, and 3 years. GEO, Gene Expression Omnibus; ROC, receiver operating characteristic

Log-rank analysis revealed a significant difference in overall survival between the two groups; the high-risk group exhibited a stronger trend towards worse overall survival (*p* = 0.018; Fig. 5A). In addition, the results of the ROC curve analysis showed that the risk model had a good prognostic effect for LUSC; the AUC values for 1-, 2-, and 3-year overall survival were 0.662, 0.662, and 0.655, respectively (Fig. 5B). The results of the validation analysis verified that the survival trend of patients stratified according to the median risk score was consistent between the GSE43131 and TCGA. The LUSC patients in the high-risk group showed poor overall survival (*p* = 0.00017, Fig. 6A). The ROC curves showed considerable prognostic capacity; the AUC values for 1-, 2-, and 3-year overall survival were 0.724, 0.724, and 0.722, respectively (Fig. 6B).

**Fig. 7:**
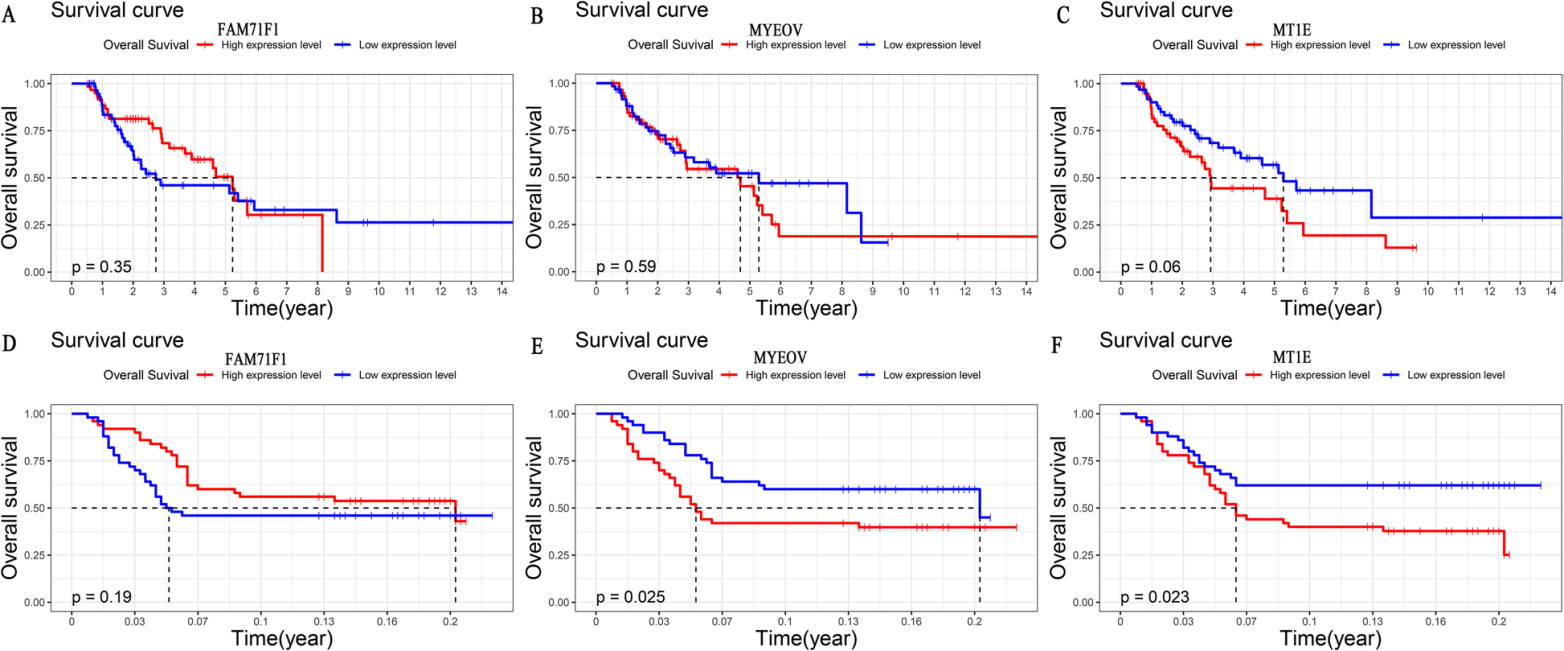
Kaplan–Meier overall survival analyses for lung squamous cell cancer patients with high or low expression levels of FAM71F1, MT1E, and MYEOV from TCGA dataset and the GEO external validation dataset. **A**–**C** Kaplan–Meier overall survival analyses based on TCGA dataset. **D**–**E** Kaplan–Meier overall survival analyses based on the GEO external validation dataset. GEO, Gene Expression Omnibus; TCGA, The Cancer Genome Atlas

We also analyzed the prognostic performance of the three m6A-related genes included in the risk model. The results showed that the impact of individual genes on the survival of LUSC patients either in TCGA or external validation dataset varied (Fig. 7). The overall survival of LUSC patients showed a similar survival trend in the two datasets. Of note, LUSC patients with a high expression level of *MT1E* were associated with poorer overall survival, which reached statistical significance both in the two datasets (TCGA: *p* = 0.06; GSE43131: *p* = 0.023, Fig. 7).

Lastly, we verified whether the risk model was an independent prognostic risk factor for LUSC patients using univariate and multivariate Cox proportional hazard analyses. The prognostic association of the risk model was maintained both in the univariate and multivariate analyses, indicating that the risk model was independently associated with the prognosis of LUSC patients, which was a risk factor for LUSC (univariate, HR: 1.90 95% confidence interval [CI]: 1.10–3.28, *p* = 0.020; multivariate, HR: 2.44, 95% CI: 1.21–3.56, *p* = 0.029) (Table 4). The results also demonstrated that cancer status (HR: 3.20, 95% CI: 1.65–6.21, *p* = 0.000) and T stage (HR: 1.95, 95% CI: 1.30–2.45, *p* = 0.017) were independent prognostic factors of risk while and sex was an independent prognostic factor of protection (HR: 0.18, 95% CI: 0.06–1.22, *p* = 0.000) in patients with LUSC (Table 4).

**Table 4:** Cox regression analyses for overall survival in patients with LUSC from TCGA

	Univariate Cox		Multivariate Cox	
	HR(95%CI)	*p*-value	HR(95%CI)	*p*-value
Risk score	1.90(1.10~3.28)	0.020*	2.44(1.21~3.56)	0.029*
Sex	0.53(0.28~1.04)	0.050*	0.18(0.06~1.22)	0.000*
Cancer status	3.72(1.93~7.16)	0.000*	3.20(1.65~6.21)	0.000*
T stage	1.37(0.98~1.94)	0.079	1.95(1.30~2.45)	0.017*
Age	1.05(0.59~1.87)	0.878		
Race	0.37(0.09~1.56)	0.116		
Smoking history	0.97(0.13~7.03)	0.974		
Pathologic stage	1.25(0.93~1.67)	0.156		
N stage	1.24(0.88~1.73)	0.236		
M stage	0.60(0.11~3.09)	0.475		

*CI* confidence interval, *HR* hazard ratio, *LUSC* lung squamous cell cancer, *TCGA* The Cancer Genome Atlas

*p < 0.05

### Three types of immune cells with different degrees of infiltration between the high- and low-risk score groups

**Fig. 8:**
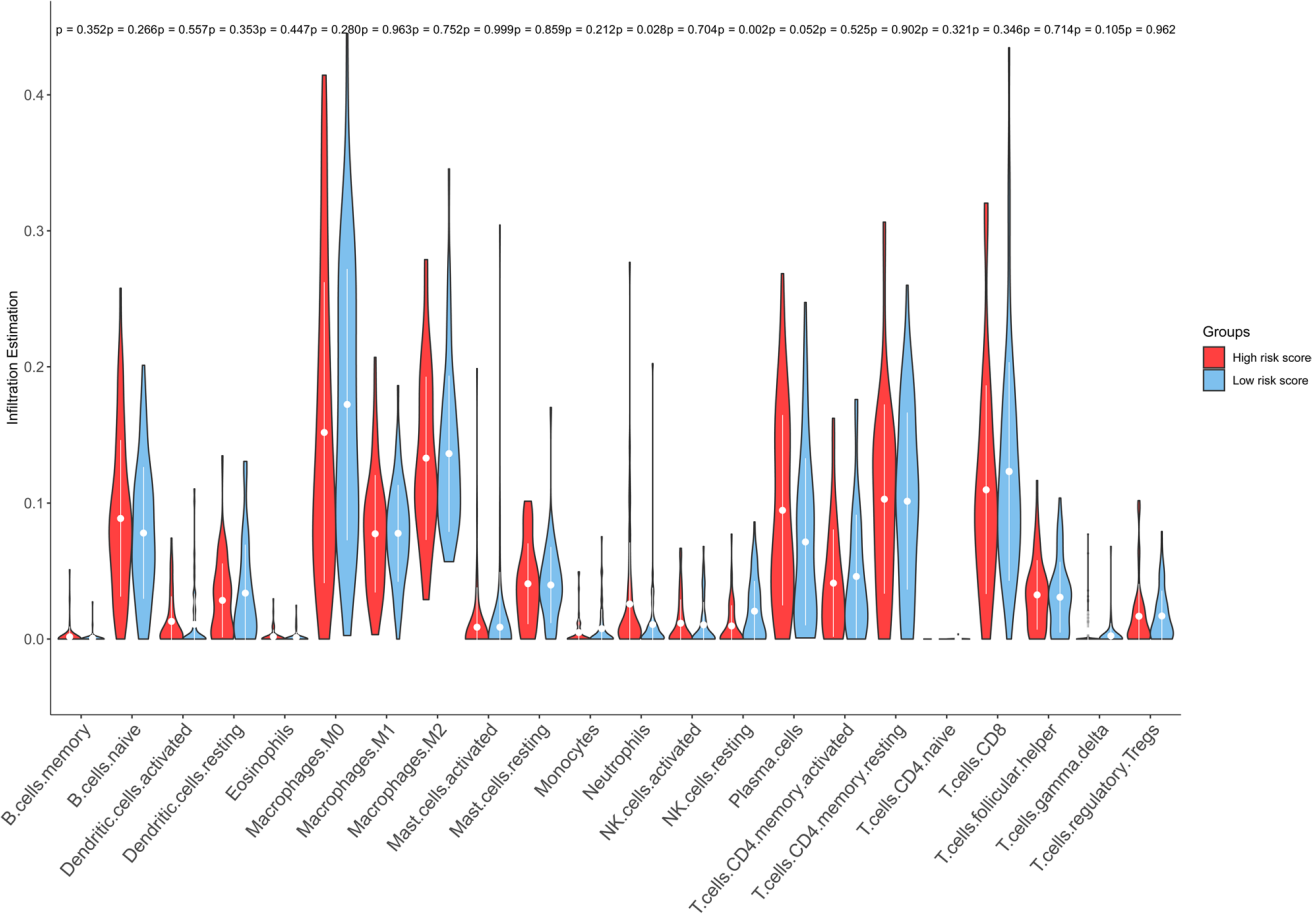
Violin plot for the differences in tumor-associated immune cell infiltrations between the high- and low-risk score subgroups

Using the TCGA data, the CIBERSORT method correctly inferred the relative infiltration signatures of LM22 in LUSC. We observed that the relative infiltration of two immune cell populations exhibited significant difference between the high- and low-risk groups. These two types of immune cells were neutrophils (*p* = 0.028) and resting NK cells (*p* = 0.002) (Fig. 8). Neutrophils showed significantly higher expression levels in the high-risk group than in the low-risk group, whereas resting NK cells exhibited significantly lower expression levels. Based on this finding, we concluded that neutrophils were negatively associated with the overall survival of LUSC patients. In contrast, resting NK cells were positively associated with the overall survival in this setting.

### Function annotation of m6A-related genes

**Fig. 9:**
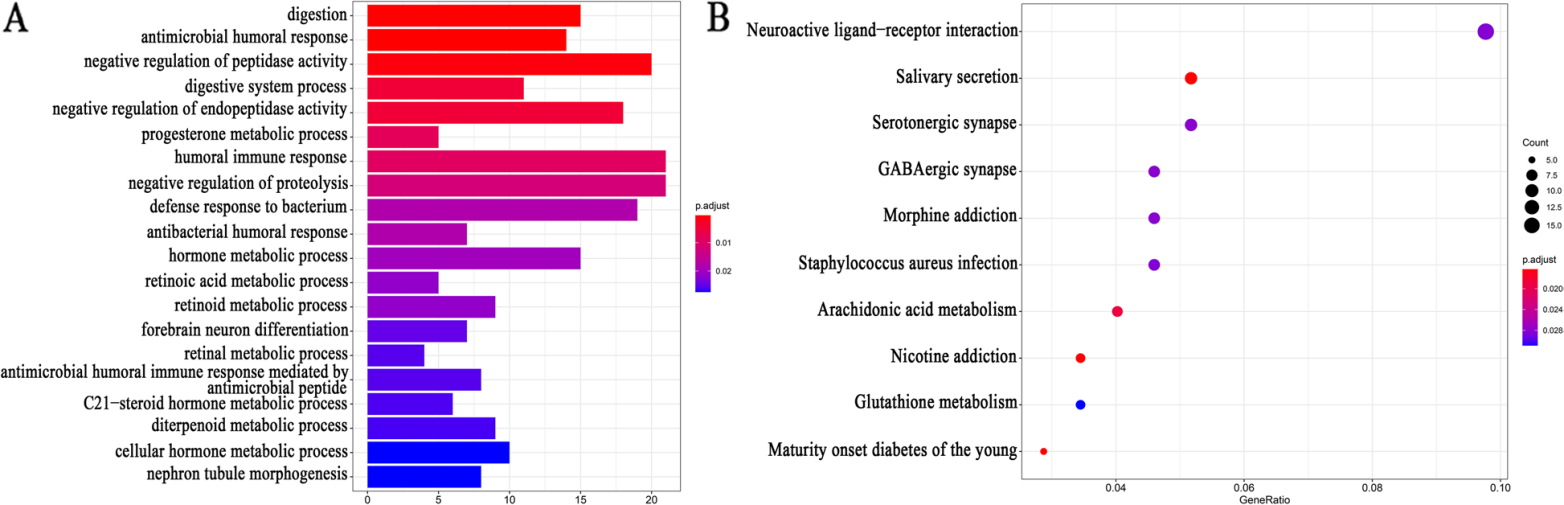
Function annotation of m6A-related genes. **A** The most significant 20 items in the GO enrichment analysis. **B** The most significant 10 items in the KEGG pathway analysis. GO, Gene Ontology; KEGG, Kyoto Encyclopedia of Genes and Genomes

We conducted function enrichment analyses to better understand the potential biological functions of m6A-related genes in the pathogenesis of LUSC. Significant terms identified by the GO analysis were negative regulation of peptidase activity, negative regulation of endopeptidase activity, humoral immune response, antimicrobial humoral response, etc. (Fig. 9A). The KEGG enrichment analysis showed that the mainly enriched pathway included nicotine addiction, maturity-onset diabetes of the young, arachidonic acid metabolism, etc. (Fig. 9B).

## Discussion

The present study definitively clarified that m6A modulators with genetic alterations (mutation and/or CNV) were strongly related to the overall survival of LUSC patients; Patients with genetic alterations were linked to longer overall survival than those without genetic alterations. Using TCGA and GSE43131 external validation datasets, we positively trained and validated a powerful prognostic risk model for LUSC, which is based on three m6A-related genes (*FAM71F1*, *MT1E*, and *MYEOV*). The risk model was an independent risk prognostic factor for LUSC; LUSC patients in the high-risk group tended towards worse overall survival compared with those in the low-risk group. In addition, we discovered that neutrophils and resting NK cells were the most likely active immune cells to influence the overall survival of LUSC patients in the high- and low-risk groups.

In distinct malignant tumors, the forms of mutation and/or CNV of m6A modulators varied. Similar to head and neck squamous cell carcinoma [22], we found that “readers” exhibited a higher frequency of alterations than “writers” and “erasers” in LUSC. This result indicated that “readers” may play more important roles than “writers” and “erasers” in these two types cancer; “erasers” may play a more vital role than the other two types of modulators in acute myeloid leukemia, breast cancer, and glioblastoma [23–25]. In liver cancer, “writers” was the most common type of alteration of modulators [26]. This observation implied that the function of m6A modulators was tumor tissue-specific. Further investigation in rigorous clinical trials of various types of malignant tumors is warranted to confirm this conclusion.

Genetic alterations of m6A modulators alter their original biological function and subsequently promote or inhibit the occurrence, progression, and metastasis of malignant tumors. Accumulating research evidence suggests that genetic variants in m6A modulators are closely related to the clinical prognosis of patients with malignant tumors. In pancreatic cancer and acute myeloid leukemia, patients with alterations of m6A modulators were associated with markedly worse overall survival versus those without alterations [27, 28]. In head and neck squamous cell carcinoma and clear cell renal cell carcinoma, there was no significant difference in the overall survival of patients with and without alterations of m6A modulators. Notably, CNV of m6A modulators could indirectly influence the overall survival of these patients by affecting the expression of m6A modulators [22, 29]. Consistent with previous studies, we also demonstrated that genetic alterations of m6A modulators were significantly associated with survival in LUSC patients; the presence of altered m6A modulators in LUSC patients predicted superior overall survival.

In recent years, an increasing number of studies have been focused on the underlying correlation of genetic alterations of m6A modulators with the prognosis of malignant tumors. It has been verified that the single nucleotide polymorphism (SNP) of staphylococcal nuclease and tudor domain containing 1 (SND1) rs118049207 is significantly associated with the risk of colorectal cancer. The levels of SND1 mRNA were obviously elevated in colorectal tumor tissues. This increase led to the downregulation of the levels of m6A modification in colorectal cancer cells to promote the risk of colorectal cancer [30]. SNP rs2416282 was obviously associated with the susceptibility of esophageal cancer by affecting YTHDC2 expression. Deletion of YTHDC2 promoted the proliferation of esophageal cancer cells through various cancer-related signaling pathways [31]. The rs7495G allele increased the risk of pancreatic cancer by promoting the expression of heterogeneous nuclear ribonucleoprotein C (hnRNPC), which is a m6A reader [32]. SNP rs5746136 of superoxide dismutase 2 in m6A modulators was strongly associated with the risk of bladder cancer. rs5746136 regulated the expression levels of SOD2 by guiding the binding of hnRNPC to SOD2, which acted as a critical tumor suppressor by promoting apoptosis and inhibiting the proliferation, metastasis, and invasion of bladder cancer cells [33]. Thus, genetic alterations of m6A modulators provide novel biomarkers for predicting cancer prognosis. However, further investigation and clinical verification are warranted.

In this study, we selected three m6A-related genes (*FAM71F1*, *MT1E*, and *MYEOV*) to generate an effective prognostic risk model for predicting the overall survival of LUSC patients. Apart from sex, cancer status, and T stage, the risk model was consistently an independent prognostic risk factor for LUSC. *MT1E* is a function isoform of metallothionein (MT) 1. Powerful evidence has indicated that MTs play a pivotal role in malignant tumor formation, growth, migration, metastasis, and drug resistance, and can be utilized for tumor diagnosis and therapy [34, 35]. MTs control the cellular homeostasis of zinc/copper, which is essential for cell differentiation and proliferation and act as antioxidants to protect cells against free radical and oxidative stress generated by antitumor drugs and radiation [36–38]. MTs can bind to mercury, cadmium, platinum, or other similar heavy metals to protect cells against heavy metal toxicity [39]. They also play protective roles against DNA damage and apoptosis [40–42]. It has been shown that *MYEOV* contributes to tumorigenesis in several types of malignant tumors [43–45]. Research has shown that *MYEOV* was specifically expressed in NSCLC and represented poor prognosis in this disease. It functioned as an amplified competing endogenous RNA in promoting metastasis through activation of the TGF-β pathway [46]. We also found that LUSC patients with high expression of *MYEOV* in the GEO external validation dataset had inferior overall survival. Thus far, the relationships of *FM71F1* with malignant tumors have only been reported in bioinformatics studies [47–49]. Hence, there is a lack of experimental investigations. Additional in vivo or in vitro research is necessary to examine the underlying association of these three m6A-related genes in LUSC.

A close connection between the immune microenvironment of tumors and overall survival has been established. Tumor-associated immune cell infiltrations represent great promising candidates for prognostic markers in malignant tumors [50, 51]. Tumor-associated neutrophils emerged as significant negative predictors of survival for breast and lung adenocarcinomas, while resting NK cells predicted adverse survival outcomes in lung cancer [52–54]. In concordance with these reports, we found that low infiltration of neutrophils can confer favorable overall survival in LUSC, where low infiltration of resting NK cells was linked to poor overall survival in LUSC. The present result contributes to the investigation of the multivariate relationships between the immune microenvironment of tumors and overall survival in patients with malignant tumors.

## Conclusions

We investigated the genetic alterations of 19 m6A modulators in LUSC and found LUSC patients with genetic alterations of m6A modulators predicted superior overall survival. Consequently, we constructed an effective prognostic risk model based on three m6A-related genes (*FAM71F1*, *MT1E*, and *MYEOV*). However, the present study was limited to bioinformatics analyses; further studies on a large sample size will be needed to demonstrate this conclusion in a clinical experiment.

## Supplementary Information

**Additional file 1 **: **Supplement Table 1**. 872 m6A related genes compared altered LUSC with unaltered LUSC in TCGA.

**Additional file 2 **: **Supplement Table 2**. Results of univariate Cox analysis in TCGA LUSC.

**Additional file 3 **: **Supplementary Table 3**. differently expressed genes between LUSC and normal tissues in TCGA dataset.

## Data Availability

All data generated and/or analyzed during this study are available from TCGA (https://www.cancer.gov, LUSC project) and a GEO external validation dataset (https://www.ncbi.nlm.nih.gov/, GSE43131).

## Abbreviations

m6A: N6-Methyladenosine
NSCLC: Non-small-cell lung cancer
LUSC: Lung squamous cell cancer
TNM: Tumor-node-metastasis
TCGA: The Cancer Genome Atlas
GEO: Gene Expression Omnibus
FPKM: Fragments per kilobase of exon model per million reads mapped
CNV: Copy number variation
LASSO: Least absolute shrinkage and selection operator
ROC: Receiver operating characteristic
AUC: Area under the curve
HR: Hazard ratio
CI: Confidence interval
TPM: Transcripts per million
CIBERSORT: Cell-type Identification By Estimating Relative Subsets Of known RNA Transcripts
LM22: 22 leukocyte subsets
GO: Gene Ontology enrichment
KEGG: Kyoto Encyclopedia of Genes and Genomes
