# Supplementary material for: Construction and validation of a novel prognostic model for lung squamous cell cancer based on N6-methyladenosine-related genes
Source: World J Surg Oncol. 2022 Feb 27;20:59. doi: 10.1186/s12957-022-02509-1 (PMC8883700; doi:10.1186/s12957-022-02509-1)
Supplement: Supplementary file 1 — Additional file 1 : Supplement Table 1. 872 m6A related genes compared altered LUSC with unaltered LUSC in TCGA. [file 12957_2022_2509_MOESM1_ESM.docx]

| supplement table 1. 872 m6A related genes compared altered LUSC with unaltered LUSC in TCGA | | | | |
| --- | --- | --- | --- | --- |
| Gene symbol | baseMean | log2FoldChange | pvalue | padj |
| *GCLC* | 16294.41 | 1.1765 | 0.00000002 | 0.00000770 |
| *WNT16* | 169.1484 | -1.8014 | 0.00000017 | 0.00004510 |
| *ZMYND10* | 229.8601 | -1.17839 | 0.00002010 | 0.00157483 |
| *THSD7A* | 381.4087 | 1.519905 | 0.00000001 | 0.00000352 |
| *SOX8* | 51.41265 | -1.67411 | 0.00000000 | 0.00000082 |
| *USH1C* | 167.8678 | 1.083977 | 0.00100838 | 0.02076160 |
| *SCIN* | 1235.59 | 1.568914 | 0.00000000 | 0.00000190 |
| *NOS2* | 2074.626 | 2.055636 | 0.00000005 | 0.00001730 |
| *TKTL1* | 127.9557 | 4.201164 | 0.00000000 | 0.00000000 |
| *SELE* | 425.4623 | -1.18828 | 0.00007400 | 0.00385655 |
| *DLEC1* | 228.6338 | 1.284953 | 0.00000811 | 0.00081708 |
| *CLCA4* | 1478.599 | -1.24912 | 0.00267943 | 0.03790273 |
| *SLC38A5* | 2639.445 | -1.0221 | 0.00000127 | 0.00021213 |
| *CYP24A1* | 1837.557 | -1.33448 | 0.00008710 | 0.00434155 |
| *ABCC2* | 351.0892 | 1.981649 | 0.00000000 | 0.00000203 |
| *GCLM* | 9612.071 | 1.154828 | 0.00000023 | 0.00005810 |
| *TLL1* | 181.5002 | -1.07889 | 0.00000026 | 0.00006320 |
| *C6* | 72.13599 | -1.17778 | 0.00033091 | 0.01045183 |
| *MAGEC2* | 267.1945 | 1.874538 | 0.00315753 | 0.04200212 |
| *COL23A1* | 174.3013 | -1.00651 | 0.00002380 | 0.00176887 |
| *RASGRF1* | 359.2836 | -1.35494 | 0.00000049 | 0.00010150 |
| *ARSF* | 14.99425 | -1.37721 | 0.00035839 | 0.01105226 |
| *ROPN1* | 8.078606 | -1.32539 | 0.00341265 | 0.04374190 |
| *ADAM7* | 1.764839 | 1.54072 | 0.00294954 | 0.04026595 |
| *CYP2W1* | 238.287 | 1.180283 | 0.00167728 | 0.02840533 |
| *MOV10L1* | 75.65501 | 1.242184 | 0.00000000 | 0.00000205 |
| *PANX2* | 1770.692 | 1.080966 | 0.00000602 | 0.00066711 |
| *ADD2* | 1780.81 | 1.175239 | 0.00004600 | 0.00281275 |
| *FGF4* | 4.186926 | -4.05555 | 0.00000039 | 0.00008620 |
| *CAPN6* | 177.4645 | -1.71414 | 0.00000006 | 0.00001990 |
| *DCX* | 8.043329 | -1.86507 | 0.00018111 | 0.00707293 |
| *FGF20* | 9.950353 | -2.5775 | 0.00000000 | 0.00000001 |
| *DNAAF6* | 23.69717 | -1.61628 | 0.00005500 | 0.00316030 |
| *AFP* | 36.154 | 1.471414 | 0.00009940 | 0.00473126 |
| *LINC01587* | 12.76517 | -1.9338 | 0.00000000 | 0.00000028 |
| *WNT11* | 732.1694 | 1.256193 | 0.00006390 | 0.00349841 |
| *CHGB* | 260.9181 | 1.000866 | 0.00271078 | 0.03815610 |
| *FETUB* | 534.4389 | 2.10322 | 0.00000220 | 0.00032809 |
| *CPA1* | 4.304289 | -1.05852 | 0.00106458 | 0.02139079 |
| *GABRP* | 1769.219 | -1.65693 | 0.00000645 | 0.00069686 |
| *KRT31* | 711.9434 | 1.31173 | 0.00049040 | 0.01356234 |
| *NXPE1* | 0.889491 | -1.31599 | 0.00189464 | 0.03050396 |
| *PGC* | 847.0222 | -1.35828 | 0.00014301 | 0.00598858 |
| *SERPIND1* | 183.3768 | -1.74138 | 0.00000031 | 0.00007300 |
| *SEC14L3* | 10.37007 | -1.25911 | 0.00019834 | 0.00749135 |
| *SLC5A1* | 343.1289 | -1.19273 | 0.00020375 | 0.00761043 |
| *TPTEP1* | 529.9294 | 1.283244 | 0.00016977 | 0.00677247 |
| *NEFH* | 218.2145 | 2.052049 | 0.00000000 | 0.00000049 |
| *RASD2* | 558.5952 | -1.00583 | 0.00001260 | 0.00112043 |
| *PVALB* | 60.88907 | 1.399785 | 0.00000730 | 0.00076323 |
| *COCH* | 1072.114 | 1.053764 | 0.00022058 | 0.00803710 |
| *TRIM9* | 298.3515 | 1.341277 | 0.00000128 | 0.00021269 |
| *CHGA* | 114.9807 | 1.213857 | 0.00032548 | 0.01032646 |
| *DHRS2* | 355.5455 | 1.182818 | 0.00047199 | 0.01328182 |
| *SPEF1* | 53.90991 | -1.10372 | 0.00000496 | 0.00058411 |
| *BPI* | 19.52922 | -1.39205 | 0.00000040 | 0.00008690 |
| *GABRE* | 3095.509 | 1.231564 | 0.00000006 | 0.00002010 |
| *OLFM4* | 173.4097 | -1.11265 | 0.00275090 | 0.03852133 |
| *NECAB2* | 192.5545 | 1.340619 | 0.00009660 | 0.00467948 |
| *DKK4* | 86.34451 | -3.10896 | 0.00000000 | 0.00000000 |
| *NEFM* | 63.20463 | 1.558051 | 0.00022044 | 0.00803710 |
| *COMP* | 1126.774 | -1.07467 | 0.00017173 | 0.00683497 |
| *ATP4A* | 64.28463 | 1.266681 | 0.00082113 | 0.01847951 |
| *GAPDHS* | 1.350206 | 1.20381 | 0.00058684 | 0.01506275 |
| *TFPI2* | 923.8686 | -1.02923 | 0.00145676 | 0.02611788 |
| *CRHR2* | 48.39429 | 1.238151 | 0.00001130 | 0.00103391 |
| *NPTX2* | 639.8342 | -1.29716 | 0.00004930 | 0.00293231 |
| *NOBOX* | 21.33546 | 2.499625 | 0.00346142 | 0.04403897 |
| *GCK* | 55.62547 | 1.060639 | 0.00009700 | 0.00468212 |
| *OGN* | 153.4896 | -1.30364 | 0.00000097 | 0.00017196 |
| *FGF8* | 14.51158 | 1.047795 | 0.00157338 | 0.02731317 |
| *KRT23* | 1872.357 | -1.53146 | 0.00001360 | 0.00119144 |
| *ODAM* | 43.87816 | -1.21295 | 0.00083955 | 0.01868567 |
| *UCP1* | 7.930386 | 1.613161 | 0.00000127 | 0.00021213 |
| *CCND1* | 16599.14 | -1.28346 | 0.00000001 | 0.00000537 |
| *CALCA* | 132.5194 | 4.174317 | 0.00000000 | 0.00000000 |
| *RFX4* | 10.79164 | 1.614454 | 0.00007530 | 0.00391164 |
| *RSPH4A* | 117.7884 | -1.14853 | 0.00000520 | 0.00059142 |
| *C6orf118* | 31.79685 | -1.36931 | 0.00046914 | 0.01324350 |
| *SEMA5A* | 1551.23 | -1.25724 | 0.00000014 | 0.00004030 |
| *HAND1* | 5.312689 | -1.40345 | 0.00263505 | 0.03752343 |
| *CDH6* | 477.1653 | -1.04194 | 0.00000757 | 0.00078589 |
| *SLC27A6* | 41.04767 | -1.61047 | 0.00000359 | 0.00046635 |
| *KNG1* | 5.127752 | 1.339341 | 0.00002890 | 0.00204377 |
| *HRG* | 34.19088 | 1.621084 | 0.00000296 | 0.00040283 |
| *HGD* | 97.36853 | 1.130129 | 0.00125274 | 0.02371552 |
| *RBP1* | 6543.169 | -1.20334 | 0.00000007 | 0.00002360 |
| *ABCC5* | 19354.96 | 1.069391 | 0.00000003 | 0.00001380 |
| *GCG* | 2.48564 | 4.073513 | 0.00007730 | 0.00399198 |
| *EFHD1* | 696.9973 | -2.40319 | 0.00000000 | 0.00000000 |
| *WNT6* | 54.25233 | -1.36809 | 0.00001120 | 0.00103391 |
| *SLC5A7* | 178.5429 | 1.78285 | 0.00018015 | 0.00707293 |
| *DLX2* | 61.17085 | -1.03268 | 0.00093214 | 0.01989964 |
| *ACTL8* | 46.15513 | 2.155705 | 0.00000001 | 0.00000549 |
| *FMO6P* | 192.2434 | 1.132939 | 0.00064472 | 0.01601320 |
| *ALDH8A1* | 52.53326 | 1.330818 | 0.00000106 | 0.00018452 |
| *GDA* | 1195.619 | 1.048006 | 0.00079380 | 0.01814761 |
| *ECRG4* | 79.95271 | -1.13016 | 0.00027542 | 0.00929666 |
| *CPN1* | 1.533825 | 1.655887 | 0.00185849 | 0.03023254 |
| *INSL4* | 4.15216 | 2.068845 | 0.00109013 | 0.02166361 |
| *GRIA2* | 20.7291 | -1.96108 | 0.00000057 | 0.00011311 |
| *CCDC170* | 203.121 | -1.0053 | 0.00000174 | 0.00027167 |
| *CHRNA2* | 2.334105 | -1.08718 | 0.00048224 | 0.01346306 |
| *NEUROG3* | 3.221293 | 1.464438 | 0.00104540 | 0.02121688 |
| *ITIH5* | 622.7 | -1.01887 | 0.00003950 | 0.00255017 |
| *CTCFL* | 585.0742 | 2.807768 | 0.00000400 | 0.00050445 |
| *SLPI* | 20485.67 | -1.32837 | 0.00000016 | 0.00004350 |
| *MATN4* | 23.24563 | -1.05097 | 0.00026744 | 0.00907359 |
| *C20orf85* | 270.5136 | -1.57939 | 0.00017717 | 0.00698853 |
| *GRM4* | 178.3115 | -1.29261 | 0.00006070 | 0.00339412 |
| *EREG* | 441.3929 | -2.10063 | 0.00000002 | 0.00000841 |
| *TMEM255A* | 373.1115 | 1.094784 | 0.00000436 | 0.00053317 |
| *CSTL1* | 9.183473 | 1.039192 | 0.00018359 | 0.00712274 |
| *PCSK2* | 167.2088 | 2.013079 | 0.00000353 | 0.00046318 |
| *GDF5* | 38.97652 | -1.32149 | 0.00000369 | 0.00047475 |
| *KRT36* | 16.25893 | -1.01292 | 0.00009090 | 0.00449391 |
| *CSN1S1* | 5.574889 | 3.966064 | 0.00004480 | 0.00277495 |
| *STATH* | 18.75435 | 1.887485 | 0.00057005 | 0.01484404 |
| *WNK4* | 105.6488 | 1.006265 | 0.00096999 | 0.02024671 |
| *PRKCG* | 16.67495 | -1.07036 | 0.00051688 | 0.01395612 |
| *OMG* | 122.8645 | -1.06148 | 0.00005460 | 0.00314725 |
| *MASP1* | 228.0235 | -1.14083 | 0.00000843 | 0.00083383 |
| *RFPL1* | 2.22138 | 1.204678 | 0.00122696 | 0.02346626 |
| *IGLL1* | 37.59249 | -1.49931 | 0.00001150 | 0.00104858 |
| *LRRC17* | 632.031 | -1.08867 | 0.00011423 | 0.00515634 |
| *BBOX1* | 194.3733 | -1.21529 | 0.00000118 | 0.00020235 |
| *PLPPR3* | 79.09058 | -2.3138 | 0.00000000 | 0.00000000 |
| *HRC* | 30.53252 | -1.00343 | 0.00000823 | 0.00082219 |
| *H19* | 3883.334 | -1.31397 | 0.00028816 | 0.00955323 |
| *TEX101* | 75.21011 | -1.09999 | 0.00054781 | 0.01445169 |
| *NAPSA* | 8436.69 | -1.0865 | 0.00060267 | 0.01530217 |
| *PPP1R1B* | 867.2986 | -1.29347 | 0.00000693 | 0.00073534 |
| *DDC* | 103.2639 | 2.089564 | 0.00000000 | 0.00000105 |
| *RGS22* | 61.0519 | -1.06941 | 0.00000874 | 0.00085557 |
| *HAPLN2* | 12.87215 | 1.117315 | 0.00047134 | 0.01327399 |
| *VSTM2L* | 486.9138 | -1.36513 | 0.00000017 | 0.00004500 |
| *KANK4* | 293.0646 | -1.66167 | 0.00000080 | 0.00014608 |
| *CCNA1* | 726.0343 | 1.335419 | 0.00006530 | 0.00354157 |
| *IRS4* | 90.29179 | 1.722638 | 0.00013123 | 0.00564518 |
| *NTS* | 24137.71 | 1.306881 | 0.00380734 | 0.04651949 |
| *TEX15* | 86.18317 | 1.365456 | 0.00070211 | 0.01682389 |
| *GSTM3* | 10514.51 | 1.570083 | 0.00000000 | 0.00000215 |
| *CHIA* | 55.59396 | -1.22894 | 0.00141977 | 0.02572034 |
| *MYCN* | 220.9287 | -1.13508 | 0.00036335 | 0.01117684 |
| *PRH2* | 13.4746 | -1.28212 | 0.00069466 | 0.01671077 |
| *FOLH1B* | 14.38734 | 1.237167 | 0.00118392 | 0.02289080 |
| *TCN1* | 607.6663 | -1.58771 | 0.00004780 | 0.00287113 |
| *HRK* | 83.7643 | -1.07758 | 0.00022298 | 0.00809046 |
| *FAM71F1* | 45.75416 | 1.890289 | 0.00000078 | 0.00014457 |
| *CGA* | 23.07148 | 3.339816 | 0.00000000 | 0.00000162 |
| *LMO2* | 647.325 | -1.06443 | 0.00000000 | 0.00000009 |
| *TBR1* | 5.450957 | 1.286012 | 0.00170526 | 0.02867305 |
| *TMOD1* | 266.9733 | 1.455309 | 0.00000012 | 0.00003620 |
| *WDR38* | 95.91211 | -1.32332 | 0.00004660 | 0.00284371 |
| *GABBR2* | 47.86194 | -1.38428 | 0.00000038 | 0.00008550 |
| *KIAA0319* | 505.5198 | 1.429888 | 0.00000976 | 0.00092733 |
| *FGFBP2* | 1068.199 | 2.012296 | 0.00000426 | 0.00052691 |
| *SLCO5A1* | 104.1406 | -1.18796 | 0.00000016 | 0.00004350 |
| *CFAP300* | 189.6292 | -1.03194 | 0.00000016 | 0.00004300 |
| *MMP13* | 6072.703 | -3.04574 | 0.00000000 | 0.00000000 |
| *PLCZ1* | 4.324848 | 1.481277 | 0.00326269 | 0.04263394 |
| *COL2A1* | 238.1629 | -1.54845 | 0.00002680 | 0.00193803 |
| *ASCL1* | 67.90311 | 3.425678 | 0.00000000 | 0.00000000 |
| *RNF113B* | 0.88619 | 1.49724 | 0.00051386 | 0.01390922 |
| *DUOXA2* | 180.5835 | -1.2455 | 0.00005630 | 0.00320511 |
| *ADAMTS18* | 127.3047 | -1.4866 | 0.00000611 | 0.00067302 |
| *OSGIN1* | 3685.299 | 1.175836 | 0.00006250 | 0.00345287 |
| *MYOCD* | 39.47721 | -1.01622 | 0.00004860 | 0.00290895 |
| *DPEP3* | 33.7959 | 1.992259 | 0.00000079 | 0.00014564 |
| *GREB1L* | 140.4904 | 1.108489 | 0.00003920 | 0.00254434 |
| *SLC14A1* | 111.2154 | -1.14324 | 0.00018096 | 0.00707293 |
| *SLC6A3* | 35.31211 | 1.198676 | 0.00021293 | 0.00786393 |
| *TM4SF5* | 3.199824 | 2.761509 | 0.00011568 | 0.00520165 |
| *FCN3* | 440.6472 | -1.22789 | 0.00000014 | 0.00004020 |
| *ADCY10* | 232.4213 | 1.095975 | 0.00137949 | 0.02521023 |
| *HHIPL2* | 73.47234 | 1.127401 | 0.00126419 | 0.02383008 |
| *S100A8* | 31197.02 | -1.02359 | 0.00407916 | 0.04862525 |
| *S100A7* | 4277.825 | -1.9161 | 0.00000267 | 0.00037514 |
| *PKLR* | 5.04832 | 1.593597 | 0.00000391 | 0.00049851 |
| *SLC4A10* | 13.00316 | 1.102024 | 0.00031196 | 0.01002512 |
| *STXBP5L* | 214.537 | 1.218137 | 0.00162754 | 0.02784314 |
| *VWA5B2* | 92.44814 | 1.053846 | 0.00003280 | 0.00221940 |
| *SLC10A6* | 116.0119 | -1.00417 | 0.00049328 | 0.01361010 |
| *ROPN1L* | 94.13879 | -1.3359 | 0.00000096 | 0.00017052 |
| *SHISAL2B* | 7.206912 | 1.00082 | 0.00097040 | 0.02024671 |
| *BHMT* | 21.39853 | 1.062764 | 0.00044647 | 0.01278776 |
| *ADAMTS19* | 14.95957 | -1.92658 | 0.00002720 | 0.00195970 |
| *SPINK7* | 28.77029 | -1.63452 | 0.00031174 | 0.01002512 |
| *CPLX2* | 175.8347 | 2.603274 | 0.00000003 | 0.00001210 |
| *TCTE1* | 30.41991 | -1.04848 | 0.00030582 | 0.00991653 |
| *NXF3* | 29.70402 | -1.8045 | 0.00000000 | 0.00000000 |
| *GPC3* | 10288.48 | 1.172222 | 0.00008600 | 0.00430762 |
| *CHRNA6* | 28.8453 | -1.32946 | 0.00000590 | 0.00065768 |
| *NTRK2* | 25078.83 | 1.268351 | 0.00004260 | 0.00269940 |
| *PLPPR1* | 390.8224 | 1.438056 | 0.00000067 | 0.00012976 |
| *CRB2* | 30.91143 | -1.2443 | 0.00000908 | 0.00087251 |
| *CACNA1B* | 704.4082 | 1.024682 | 0.00074436 | 0.01745768 |
| *HABP2* | 136.4611 | -1.54528 | 0.00000032 | 0.00007540 |
| *SCGB1A1* | 3940.824 | -1.42922 | 0.00027896 | 0.00935790 |
| *CDH22* | 85.95052 | -1.60025 | 0.00015617 | 0.00634446 |
| *SPATA4* | 12.77636 | -1.01802 | 0.00046946 | 0.01324350 |
| *ANO4* | 36.51369 | -1.06444 | 0.00020730 | 0.00772056 |
| *POU4F2* | 1.393438 | 2.004319 | 0.00187217 | 0.03032532 |
| *AKR1C2* | 39284.08 | 1.135736 | 0.00031254 | 0.01003291 |
| *GFRA1* | 199.0947 | -1.07513 | 0.00001560 | 0.00130330 |
| *BOLL* | 5.531119 | 1.331129 | 0.00001430 | 0.00123580 |
| *CAPSL* | 68.08586 | -1.12864 | 0.00080301 | 0.01825182 |
| *FAM81B* | 73.19839 | -1.27073 | 0.00003640 | 0.00239895 |
| *CIBAR2* | 131.102 | -1.12729 | 0.00003920 | 0.00254434 |
| *KCNJ16* | 158.3409 | 1.531061 | 0.00001970 | 0.00154962 |
| *CLCA3P* | 81.98108 | -1.58713 | 0.00002920 | 0.00205813 |
| *MAGEC1* | 252.7579 | 1.986652 | 0.00194191 | 0.03093046 |
| *ADCY8* | 300.3801 | 2.770708 | 0.00000005 | 0.00001790 |
| *AFF2* | 670.182 | 1.126774 | 0.00006800 | 0.00360304 |
| *MAGEA8* | 11.44608 | 1.401185 | 0.00003560 | 0.00236317 |
| *ART3* | 34.15122 | -2.29452 | 0.00000000 | 0.00000035 |
| *NAA11* | 13.85628 | 3.041038 | 0.00001280 | 0.00113645 |
| *FUT6* | 186.4531 | -1.34533 | 0.00005340 | 0.00310111 |
| *LRFN2* | 13.16144 | 1.129002 | 0.00158958 | 0.02752668 |
| *ZIC3* | 10.08307 | 1.615518 | 0.00387310 | 0.04702541 |
| *LHFPL4* | 16.01177 | 2.597586 | 0.00000000 | 0.00000004 |
| *C1orf158* | 24.01095 | -1.23728 | 0.00278036 | 0.03872835 |
| *KCNJ6* | 37.33121 | -1.4565 | 0.00000178 | 0.00027610 |
| *SLC30A2* | 15.38692 | -1.00429 | 0.00014652 | 0.00607802 |
| *TRIM63* | 6.595601 | -1.6009 | 0.00000051 | 0.00010347 |
| *HPD* | 47.1084 | 1.169865 | 0.00000058 | 0.00011512 |
| *CPA2* | 30.4619 | 1.135093 | 0.00041615 | 0.01224682 |
| *CBR1* | 14485.56 | 1.058725 | 0.00000247 | 0.00035757 |
| *ACTC1* | 14.41684 | -1.09967 | 0.00013045 | 0.00563663 |
| *PLA2G4D* | 89.18896 | -2.0199 | 0.00000000 | 0.00000000 |
| *CELF3* | 5.746946 | 1.381288 | 0.00057947 | 0.01498119 |
| *FAM131B* | 162.3437 | -1.24576 | 0.00000000 | 0.00000220 |
| *RSPH1* | 185.225 | -1.18118 | 0.00001370 | 0.00119811 |
| *FTCD* | 60.94107 | 1.224035 | 0.00035275 | 0.01094616 |
| *TMEM190* | 53.98809 | -1.1403 | 0.00048888 | 0.01353093 |
| *AZGP1* | 579.6503 | -1.59019 | 0.00000014 | 0.00004030 |
| *PTGER1* | 40.85467 | -1.07833 | 0.00001700 | 0.00138014 |
| *SCGB3A1* | 1922.783 | -1.54486 | 0.00000877 | 0.00085557 |
| *AC008132.13* | 2.547767 | 1.226006 | 0.00038459 | 0.01166781 |
| *KASH5* | 22.62531 | 1.047257 | 0.00276452 | 0.03869669 |
| *AQP5* | 364.0929 | -2.28825 | 0.00000000 | 0.00000000 |
| *LHX8* | 24.10974 | 1.413634 | 0.00313940 | 0.04190371 |
| *TRIM58* | 20.98291 | 1.701665 | 0.00000040 | 0.00008690 |
| *CAPN13* | 1152.181 | -1.22559 | 0.00005660 | 0.00321667 |
| *KCNJ3* | 11.42347 | 1.436255 | 0.00157624 | 0.02734931 |
| *NEUROD1* | 2.199488 | 2.652723 | 0.00125510 | 0.02372203 |
| *EN1* | 319.3378 | -1.28941 | 0.00043552 | 0.01261472 |
| *C1orf189* | 22.04828 | -1.38248 | 0.00001120 | 0.00103391 |
| *LENEP* | 3.577029 | 1.036098 | 0.00001760 | 0.00142270 |
| *PDCL2* | 4.845847 | 1.524732 | 0.00370563 | 0.04593893 |
| *PTX3* | 198.1368 | -1.08944 | 0.00002900 | 0.00205042 |
| *SCRG1* | 12.05309 | -1.05378 | 0.00005190 | 0.00304662 |
| *RAET1E* | 178.8098 | -1.01676 | 0.00218119 | 0.03338763 |
| *BMPER* | 162.7361 | -1.18375 | 0.00000495 | 0.00058411 |
| *SLC35G3* | 3.757495 | 1.285735 | 0.00180557 | 0.02973723 |
| *DEFA5* | 3.832088 | -3.10517 | 0.00422288 | 0.04969324 |
| *C9orf24* | 164.8389 | -1.2671 | 0.00003590 | 0.00237330 |
| *ZMAT4* | 61.6273 | 2.043172 | 0.00000198 | 0.00030050 |
| *CLDN3* | 860.0612 | -1.05028 | 0.00062376 | 0.01565540 |
| *SLC7A3* | 26.22713 | 1.655918 | 0.00000981 | 0.00092957 |
| *LRRC18* | 14.95416 | -1.0108 | 0.00034584 | 0.01077895 |
| *OTX2* | 48.70684 | 2.007251 | 0.00001080 | 0.00101112 |
| *SERPINA12* | 12.79755 | -1.40906 | 0.00166228 | 0.02824605 |
| *SVOP* | 8.533274 | 1.338969 | 0.00001160 | 0.00105004 |
| *SPIC* | 5.376228 | -1.38495 | 0.00027920 | 0.00935790 |
| *TMEM100* | 219.4368 | -1.35709 | 0.00000013 | 0.00003890 |
| *MOGAT2* | 16.56659 | -1.2161 | 0.00035966 | 0.01107307 |
| *LMO1* | 57.77387 | 1.215222 | 0.00008910 | 0.00441436 |
| *MMP10* | 8588.699 | -1.29032 | 0.00066967 | 0.01638853 |
| *HTR3A* | 71.81753 | 1.015917 | 0.00123490 | 0.02354166 |
| *STRCP1* | 20.65448 | 1.045405 | 0.00000614 | 0.00067351 |
| *MS4A8* | 176.4221 | -1.70454 | 0.00001060 | 0.00099852 |
| *OR51E2* | 12.81004 | 1.631063 | 0.00000779 | 0.00079527 |
| *NLRP7* | 161.762 | -2.02967 | 0.00000001 | 0.00000537 |
| *ATCAY* | 4.421157 | -1.21724 | 0.00102549 | 0.02098726 |
| *KLK5* | 350.8356 | -1.33461 | 0.00176999 | 0.02937027 |
| *TBX10* | 5.008349 | 1.068144 | 0.00163108 | 0.02789012 |
| *TEKT1* | 132.4843 | -1.27963 | 0.00047774 | 0.01339036 |
| *SOST* | 1922.337 | 1.40914 | 0.00242637 | 0.03589693 |
| *MOBP* | 52.04964 | 1.875634 | 0.00000001 | 0.00000680 |
| *DYNLRB2* | 42.00556 | -1.08004 | 0.00001130 | 0.00103645 |
| *ADAM18* | 2.825159 | 2.383523 | 0.00016354 | 0.00657591 |
| *MUCL3* | 33.58118 | -1.00197 | 0.00161908 | 0.02773866 |
| *WFDC12* | 34.95509 | -1.22401 | 0.00077287 | 0.01786549 |
| *SOX14* | 20.35909 | -3.34963 | 0.00000054 | 0.00010921 |
| *KLK7* | 978.6853 | -1.83028 | 0.00000348 | 0.00045929 |
| *ZBBX* | 50.67331 | -1.2577 | 0.00025322 | 0.00880883 |
| *PARM1* | 3815.725 | -1.34942 | 0.00000000 | 0.00000125 |
| *RAB3B* | 1193.731 | 1.554828 | 0.00000002 | 0.00000758 |
| *SCN9A* | 1523.858 | 1.189646 | 0.00003720 | 0.00242727 |
| *SLC38A11* | 185.8704 | 1.862267 | 0.00000254 | 0.00036348 |
| *BCRP2* | 9.150939 | 1.214643 | 0.00016294 | 0.00657415 |
| *MT1E* | 1852.099 | -1.09376 | 0.00000141 | 0.00022934 |
| *FABP4* | 474.5401 | -1.57962 | 0.00000241 | 0.00035196 |
| *CST5* | 6.35208 | -1.07181 | 0.00081792 | 0.01844823 |
| *CST2* | 90.34303 | -1.03631 | 0.00011520 | 0.00518683 |
| *CST1* | 1260.724 | -1.52471 | 0.00000297 | 0.00040283 |
| *VSTM2A* | 0.99291 | -1.48376 | 0.00335145 | 0.04329443 |
| *OR5E1P* | 1.732398 | 1.118204 | 0.00322661 | 0.04241032 |
| *SYT9* | 47.28367 | 1.572014 | 0.00001600 | 0.00132620 |
| *DYDC1* | 4.580171 | -1.07882 | 0.00119560 | 0.02302289 |
| *CEL* | 1266.633 | 1.116732 | 0.00081188 | 0.01838242 |
| *PLA2G1B* | 33.16889 | -1.16636 | 0.00010405 | 0.00487730 |
| *OBP2B* | 1.757111 | -1.66954 | 0.00218984 | 0.03349105 |
| *CLDN20* | 68.17407 | 1.114003 | 0.00026412 | 0.00904552 |
| *KRT20* | 34.56893 | 1.527195 | 0.00032276 | 0.01025880 |
| *SSX6P* | 2.192795 | 1.808501 | 0.00056900 | 0.01483102 |
| *NMRAL2P* | 3583.698 | 2.044111 | 0.00000001 | 0.00000401 |
| *DEFB4A* | 43.99194 | -1.16028 | 0.00372856 | 0.04602006 |
| *SYCE1* | 8.474462 | 1.480073 | 0.00041343 | 0.01222475 |
| *CYP4F11* | 7566.883 | 1.163618 | 0.00233069 | 0.03496430 |
| *SCG2* | 301.4923 | 1.085207 | 0.00002620 | 0.00190668 |
| *B3GALT1* | 49.47212 | 1.656827 | 0.00000037 | 0.00008350 |
| *SYNPO2* | 1500.931 | -1.05948 | 0.00002120 | 0.00163461 |
| *MYEOV* | 961.8004 | -1.97937 | 0.00000004 | 0.00001520 |
| *RXFP4* | 3.104448 | -1.20268 | 0.00048510 | 0.01348514 |
| *LRRN3* | 63.42808 | -1.22603 | 0.00000003 | 0.00001230 |
| *DMRT2* | 1122.797 | 1.151783 | 0.00015542 | 0.00632126 |
| *CSPG4* | 2835.588 | -1.186 | 0.00000847 | 0.00083383 |
| *APOBEC4* | 25.356 | -1.27517 | 0.00270502 | 0.03812051 |
| *SUSD5* | 146.0608 | -1.36601 | 0.00000000 | 0.00000018 |
| *PIFO* | 320.8141 | -1.04709 | 0.00004410 | 0.00275818 |
| *CNTNAP2* | 3276.994 | 1.011989 | 0.00148718 | 0.02642681 |
| *CA5A* | 6.20717 | 1.111861 | 0.00088213 | 0.01926939 |
| *DES* | 333.9358 | -1.11695 | 0.00000778 | 0.00079527 |
| *PCSK1* | 247.9313 | 1.164618 | 0.00001940 | 0.00152863 |
| *PLEKHD1* | 45.86351 | 1.080713 | 0.00002830 | 0.00201940 |
| *KCNE5* | 12.60397 | -1.11917 | 0.00000035 | 0.00008020 |
| *SLC35G1* | 1800.576 | 1.307614 | 0.00000000 | 0.00000125 |
| *MAP3K19* | 45.66104 | -1.1821 | 0.00008480 | 0.00426500 |
| *NANOGP1* | 2.873703 | -1.53067 | 0.00013730 | 0.00578392 |
| *SOX11* | 181.4076 | -1.88091 | 0.00000212 | 0.00031821 |
| *FAM9B* | 3.912853 | 1.379323 | 0.00320044 | 0.04229525 |
| *UMODL1* | 100.0313 | 1.290287 | 0.00012763 | 0.00556215 |
| *AC011944.1* | 21.45807 | -1.27911 | 0.00000045 | 0.00009580 |
| *LCN15* | 3.731495 | 3.060512 | 0.00000117 | 0.00020235 |
| *PPP1R42* | 14.07637 | -1.20106 | 0.00008760 | 0.00435260 |
| *SPINK6* | 13.63487 | -2.15663 | 0.00000006 | 0.00001990 |
| *GLDC* | 265.5762 | 1.040295 | 0.00031353 | 0.01004662 |
| *ALOX15B* | 933.2923 | -1.03291 | 0.00001510 | 0.00127508 |
| *FCER1A* | 150.423 | -1.05646 | 0.00005690 | 0.00322050 |
| *ARL14* | 53.46743 | -1.00204 | 0.00227860 | 0.03441666 |
| *WDR97* | 124.2384 | 1.008008 | 0.00013747 | 0.00578399 |
| *FAM216B* | 153.6437 | -1.24611 | 0.00043600 | 0.01261472 |
| *C1orf194* | 67.01357 | -1.39535 | 0.00003840 | 0.00250257 |
| *C12orf40* | 1.639901 | 1.739637 | 0.00371869 | 0.04597023 |
| *KCTD4* | 3.235708 | 1.36271 | 0.00140121 | 0.02550676 |
| *MEIOC* | 71.3806 | 1.348888 | 0.00000054 | 0.00010921 |
| *AC004832.1* | 17.09592 | -1.2542 | 0.00033623 | 0.01057268 |
| *MUC16* | 1078.88 | -1.50074 | 0.00001890 | 0.00150993 |
| *FDCSP* | 949.4376 | -3.43046 | 0.00000000 | 0.00000000 |
| *ANKRD62* | 16.792 | 1.030039 | 0.00077441 | 0.01788933 |
| *NEUROG1* | 1.667365 | 2.378168 | 0.00000024 | 0.00005930 |
| *MGAT4C* | 63.47267 | 1.34544 | 0.00032720 | 0.01037177 |
| *TRIM49B* | 3.593095 | 2.629028 | 0.00144325 | 0.02594621 |
| *FAM153B* | 41.43496 | 1.022764 | 0.00199471 | 0.03147360 |
| *KCNA4* | 14.04365 | 1.38143 | 0.00099529 | 0.02059571 |
| *TMIGD1* | 0.926344 | 1.535721 | 0.00247535 | 0.03618694 |
| *RNASE10* | 36.31827 | 1.093764 | 0.00152099 | 0.02671867 |
| *RGS6* | 258.2512 | 1.453525 | 0.00000228 | 0.00033733 |
| *TSPEAR-AS2* | 103.1722 | 1.041331 | 0.00048184 | 0.01346260 |
| *CSMD1* | 229.608 | 1.057556 | 0.00209154 | 0.03245100 |
| *CABCOCO1* | 54.61518 | -1.21918 | 0.00000959 | 0.00091369 |
| *GRIN2A* | 126.2189 | -2.17938 | 0.00000000 | 0.00000125 |
| *GKN2* | 70.36109 | -1.05009 | 0.00316108 | 0.04200645 |
| *ACP7* | 384.648 | 1.422082 | 0.00000821 | 0.00082208 |
| *LRRC55* | 82.4666 | -1.51637 | 0.00000003 | 0.00001270 |
| *TREML3P* | 209.1278 | 2.393198 | 0.00000000 | 0.00000002 |
| *CNTN2* | 59.37234 | -1.75008 | 0.00000891 | 0.00086259 |
| *UMODL1-AS1* | 17.47015 | -1.303 | 0.00009290 | 0.00456764 |
| *POU3F2* | 56.61096 | -1.69908 | 0.00005260 | 0.00307939 |
| *MUC6* | 34.98445 | -1.74027 | 0.00000002 | 0.00000770 |
| *WASIR1* | 1.872419 | 1.450334 | 0.00365503 | 0.04560588 |
| *GALNT17* | 229.401 | -1.13706 | 0.00001610 | 0.00132620 |
| *CD24P4* | 11.88583 | -1.71425 | 0.00000000 | 0.00000009 |
| *HS6ST3* | 70.96963 | 2.152386 | 0.00000001 | 0.00000561 |
| *MORN5* | 44.31596 | -1.34069 | 0.00018336 | 0.00712179 |
| *KLHL34* | 6.580635 | 1.13256 | 0.00092225 | 0.01980322 |
| *NKAIN3* | 4.851658 | 1.55099 | 0.00210777 | 0.03260256 |
| *HTR3E* | 5.039622 | 1.143279 | 0.00106923 | 0.02143003 |
| *AKAP14* | 29.17909 | -1.45737 | 0.00001940 | 0.00152863 |
| *CYP4F3* | 6317.606 | 1.141916 | 0.00413078 | 0.04901435 |
| *CYP27C1* | 197.9061 | -1.11214 | 0.00018741 | 0.00722317 |
| *CFAP73* | 54.14025 | -1.01948 | 0.00019290 | 0.00736284 |
| *MPPED1* | 256.5804 | 1.33969 | 0.00178595 | 0.02951038 |
| *KRT16* | 45313.31 | -1.04179 | 0.00058240 | 0.01498119 |
| *KRTAP17-1* | 2.552107 | 2.444923 | 0.00001780 | 0.00143406 |
| *FGF3* | 11.40134 | -3.00145 | 0.00002180 | 0.00165859 |
| *AKR1C1* | 48598.3 | 1.38616 | 0.00006490 | 0.00353485 |
| *FAM9C* | 3.517041 | -2.26973 | 0.00008730 | 0.00434753 |
| *DMBT1* | 3699.893 | -1.09441 | 0.00110935 | 0.02188000 |
| *CPSF4L* | 11.59529 | -1.2183 | 0.00001540 | 0.00129280 |
| *CLCN1* | 42.14954 | -1.43495 | 0.00000024 | 0.00005930 |
| *FAM166A* | 3.318834 | 1.141468 | 0.00000356 | 0.00046461 |
| *PLA2G2A* | 215.5131 | -1.08474 | 0.00018072 | 0.00707293 |
| *JAKMIP3* | 264.0651 | 1.42017 | 0.00000010 | 0.00003120 |
| *TCTEX1D4* | 11.35822 | -1.02945 | 0.00016129 | 0.00651503 |
| *INSC* | 15.09965 | -1.05946 | 0.00002300 | 0.00172967 |
| *CFAP77* | 43.33609 | -1.04818 | 0.00093904 | 0.01990429 |
| *SNTN* | 144.349 | -1.15863 | 0.00051481 | 0.01392152 |
| *GLRA4* | 3.026594 | -1.33862 | 0.00250672 | 0.03635481 |
| *KRT77* | 206.7823 | 1.487883 | 0.00019644 | 0.00744967 |
| *AKR1C3* | 29981.05 | 1.47326 | 0.00000506 | 0.00058877 |
| *LINC00615* | 15.69054 | 1.98723 | 0.00000367 | 0.00047318 |
| *SFTA2* | 599.6458 | -1.03853 | 0.00222451 | 0.03383104 |
| *ADH7* | 9491.525 | 1.152103 | 0.00221782 | 0.03380193 |
| *MMP1* | 17706.22 | -1.29757 | 0.00000761 | 0.00078648 |
| *UGT2B15* | 10.32201 | 1.122243 | 0.00167201 | 0.02836472 |
| *AC068631.1* | 25.01577 | 1.26644 | 0.00000008 | 0.00002540 |
| *ADGRA1* | 8.390431 | -1.30097 | 0.00066562 | 0.01637148 |
| *LYPD2* | 104.9191 | -2.08947 | 0.00000019 | 0.00005010 |
| *DCHS2* | 87.99316 | -1.07255 | 0.00002790 | 0.00200246 |
| *MYH6* | 10.76541 | 1.141683 | 0.00308681 | 0.04149987 |
| *CCER1* | 2.235875 | 2.839647 | 0.00000297 | 0.00040283 |
| *TXNRD1* | 26888.18 | 1.185368 | 0.00000005 | 0.00001790 |
| *ZNF536* | 12.49827 | 1.249398 | 0.00109796 | 0.02175947 |
| *AKR1C4* | 29.43592 | 1.672537 | 0.00000150 | 0.00023977 |
| *FAM3D* | 422.631 | -1.11786 | 0.00025212 | 0.00878966 |
| *ABCA4* | 1526.948 | 1.804557 | 0.00000470 | 0.00056553 |
| *CES1* | 35006.66 | 1.524979 | 0.00003180 | 0.00216216 |
| *RORB* | 80.25184 | -1.24401 | 0.00000188 | 0.00028767 |
| *RNA5SP442* | 1.805784 | 1.56172 | 0.00055679 | 0.01462060 |
| *LINC00970* | 9.697384 | -1.45975 | 0.00045077 | 0.01287660 |
| *C10orf62* | 2.003777 | 1.025655 | 0.00160556 | 0.02763850 |
| *TCEAL5* | 27.72821 | 1.301149 | 0.00197222 | 0.03121632 |
| *CLPSL1* | 11.45361 | -1.65599 | 0.00081725 | 0.01844823 |
| *SP5* | 50.04376 | -1.70817 | 0.00000002 | 0.00000727 |
| *C9orf129* | 1.988813 | 1.235771 | 0.00113056 | 0.02215004 |
| *PKP4-AS1* | 147.7655 | 1.249824 | 0.00000446 | 0.00054415 |
| *GUCY2EP* | 13.57795 | 1.513043 | 0.00104898 | 0.02125301 |
| *C9orf135* | 30.07861 | -1.20871 | 0.00079262 | 0.01813221 |
| *NAT8B* | 21.08943 | -1.07955 | 0.00011035 | 0.00504557 |
| *FBXO47* | 3.091389 | 1.185448 | 0.00020391 | 0.00761043 |
| *BLACE* | 1.298414 | 1.103101 | 0.00281893 | 0.03897058 |
| *KRT17P7* | 1.359946 | 1.734544 | 0.00104458 | 0.02121688 |
| *KRT17P5* | 1.355619 | 1.511969 | 0.00146451 | 0.02616869 |
| *LINC01602* | 16.95514 | 2.207454 | 0.00000068 | 0.00013043 |
| *IGLL3P* | 19.63825 | -1.63626 | 0.00000517 | 0.00059142 |
| *AL359555.1* | 1.265115 | -1.48292 | 0.00005580 | 0.00319410 |
| *AC109583.1* | 257.4448 | 1.171057 | 0.00405650 | 0.04848592 |
| *XKR4* | 28.31689 | -1.78431 | 0.00000027 | 0.00006550 |
| *D87024.1* | 8.330785 | -1.20395 | 0.00051361 | 0.01390922 |
| *IGKV6-21* | 672.1252 | -1.03125 | 0.00244957 | 0.03598411 |
| *IGKV6D-41* | 5.941406 | -1.21999 | 0.00194519 | 0.03095490 |
| *IGLV2-33* | 25.05191 | -1.03358 | 0.00130245 | 0.02429086 |
| *IGHD* | 2802.282 | -1.2524 | 0.00014942 | 0.00615755 |
| *KRTAP29-1* | 4.251614 | 1.48428 | 0.00015868 | 0.00643149 |
| *KRTAP2-3* | 4.532997 | -1.85752 | 0.00002480 | 0.00182507 |
| *CGB8* | 2.128967 | -1.50347 | 0.00211913 | 0.03270676 |
| *GSTM2* | 2111.115 | 1.603982 | 0.00000000 | 0.00000001 |
| *AKR1B10P1* | 134.7656 | 1.422466 | 0.00163587 | 0.02790516 |
| *AC019080.1* | 752.5238 | 1.073751 | 0.00000611 | 0.00067302 |
| *AC007277.1* | 2.581418 | -1.05678 | 0.00110338 | 0.02179861 |
| *SLC6A10P* | 90.11972 | 1.285243 | 0.00420735 | 0.04960125 |
| *PNMA6E* | 9.876137 | 2.76563 | 0.00022770 | 0.00820463 |
| *C16orf90* | 3.251245 | 1.012668 | 0.00258631 | 0.03710210 |
| *PRSS41* | 6.508141 | 2.044859 | 0.00000504 | 0.00058877 |
| *RPS12P16* | 1.236772 | 1.073965 | 0.00147888 | 0.02634551 |
| *FAM166B* | 99.11767 | -1.02692 | 0.00003500 | 0.00232777 |
| *C5orf49* | 117.8105 | -1.45785 | 0.00000068 | 0.00013043 |
| *UBE2QL1* | 256.7412 | -1.52966 | 0.00000014 | 0.00004030 |
| *AKR1C7P* | 207.7003 | 1.340946 | 0.00002910 | 0.00205549 |
| *SKOR2* | 1.092623 | 2.400283 | 0.00183696 | 0.02998869 |
| *TPTE2P4* | 2.013225 | 3.076546 | 0.00011936 | 0.00531320 |
| *CAPNS1P1* | 2.668181 | 1.05067 | 0.00024603 | 0.00866092 |
| *AL390237.1* | 1.166567 | 1.549541 | 0.00160208 | 0.02763489 |
| *KRT8P43* | 3.629912 | 1.022887 | 0.00078215 | 0.01798097 |
| *AC008060.1* | 5.133607 | 1.738032 | 0.00334002 | 0.04319414 |
| *FAM8A6P* | 1.820451 | 2.532828 | 0.00011640 | 0.00521418 |
| *RPS3AP2* | 2.228102 | 1.061378 | 0.00047404 | 0.01332899 |
| *GSTA8P* | 154.1762 | 2.518702 | 0.00000071 | 0.00013366 |
| *LINC02860* | 1.87665 | 1.771632 | 0.00002650 | 0.00192434 |
| *PSMB11* | 0.830629 | 1.550395 | 0.00417469 | 0.04943282 |
| *AC079305.1* | 28.85446 | 1.082658 | 0.00000006 | 0.00002110 |
| *Y_RNA* | 0.843229 | 1.494252 | 0.00046680 | 0.01319872 |
| *RNA5SP111* | 5.235394 | 2.714194 | 0.00001860 | 0.00148885 |
| *IGLV9-49* | 691.1576 | -1.43647 | 0.00000490 | 0.00058206 |
| *AC005165.1* | 34.29389 | 1.008966 | 0.00076641 | 0.01780937 |
| *GSTA6P* | 0.931263 | 1.61613 | 0.00262659 | 0.03743317 |
| *AC002463.1* | 2.138713 | 2.457336 | 0.00126278 | 0.02382905 |
| *LINC02561* | 121.6059 | 1.067243 | 0.00106055 | 0.02135847 |
| *AL391427.1* | 39.4512 | 1.772312 | 0.00000000 | 0.00000205 |
| *AC114489.2* | 25.46519 | 1.16112 | 0.00003940 | 0.00254719 |
| *AL135787.1* | 3.645196 | 1.100165 | 0.00315032 | 0.04195563 |
| *LINC01823* | 1.054839 | -1.18838 | 0.00354778 | 0.04481048 |
| *AC012363.1* | 5.00207 | 1.868446 | 0.00413218 | 0.04901435 |
| *AC114812.2* | 13.90879 | 1.093596 | 0.00014737 | 0.00609189 |
| *RORB-AS1* | 1.074712 | -1.59561 | 0.00223356 | 0.03393930 |
| *LINC02470* | 1.966864 | 1.548145 | 0.00029989 | 0.00976007 |
| *LA16c-83F12.6* | 11.45594 | 1.962426 | 0.00020106 | 0.00754695 |
| *SFTA1P* | 164.2532 | -1.03341 | 0.00003690 | 0.00241468 |
| *AKR1C5P* | 3.82327 | 1.273508 | 0.00246203 | 0.03610337 |
| *RFPL1S* | 69.57586 | 1.278991 | 0.00001120 | 0.00103391 |
| *SP3P* | 2.45579 | 1.58755 | 0.00412031 | 0.04893423 |
| *AC005281.1* | 6.024993 | 1.664986 | 0.00000186 | 0.00028633 |
| *AC069281.1* | 1.554058 | 1.306473 | 0.00061974 | 0.01560585 |
| *AC009299.1* | 0.915549 | 1.999593 | 0.00096186 | 0.02016678 |
| *UBXN7-AS1* | 2.398738 | 1.119132 | 0.00002210 | 0.00167233 |
| *LGALS17A* | 32.31737 | 1.085419 | 0.00078295 | 0.01798097 |
| *ZNF503-AS1* | 92.84419 | -1.12398 | 0.00004410 | 0.00275818 |
| *AC244107.1* | 5.358699 | 1.923639 | 0.00241872 | 0.03585551 |
| *KRT16P6* | 229.8737 | -1.37354 | 0.00057201 | 0.01487230 |
| *MLXP1* | 48.43665 | 1.976907 | 0.00000249 | 0.00035988 |
| *AC019118.1* | 1.80584 | 1.13794 | 0.00037841 | 0.01153971 |
| *AC000124.1* | 2.073319 | 1.106997 | 0.00201202 | 0.03159687 |
| *LINC00161* | 6.3795 | 1.085539 | 0.00102733 | 0.02101262 |
| *LINC00629* | 7.722354 | 1.254508 | 0.00000034 | 0.00007930 |
| *LINC01153* | 1.071476 | 2.39802 | 0.00337446 | 0.04346457 |
| *ACNATP* | 5.669445 | 3.040905 | 0.00000148 | 0.00023785 |
| *AL391056.1* | 31.783 | -1.25746 | 0.00000005 | 0.00001760 |
| *CLYBL-AS2* | 0.97047 | 1.244659 | 0.00213666 | 0.03287679 |
| *SELENOOLP* | 11.43958 | 1.509982 | 0.00088027 | 0.01925256 |
| *LINC01320* | 4.48912 | -1.59892 | 0.00149473 | 0.02649437 |
| *AP000593.2* | 1.047404 | 2.06656 | 0.00141927 | 0.02572034 |
| *ARL4AP3* | 2.639495 | 1.673851 | 0.00069766 | 0.01676282 |
| *AC026355.2* | 47.02232 | 1.469348 | 0.00000071 | 0.00013366 |
| *AC005042.1* | 4.204405 | 2.01939 | 0.00000620 | 0.00067590 |
| *AC141930.1* | 5.339164 | 1.139724 | 0.00182417 | 0.02984106 |
| *ROCR* | 2.095845 | -3.01764 | 0.00000006 | 0.00001960 |
| *CES1P1* | 124.5767 | 2.112302 | 0.00000007 | 0.00002360 |
| *SRGAP3-AS2* | 59.57563 | -1.4706 | 0.00044618 | 0.01278776 |
| *AL356310.1* | 2.166393 | -1.5236 | 0.00093303 | 0.01989964 |
| *SLC47A1P1* | 3.513239 | 1.495949 | 0.00020114 | 0.00754695 |
| *ANKRD20A8P* | 2.980321 | 1.609763 | 0.00010263 | 0.00483627 |
| *AL596223.1* | 13.25367 | 2.22146 | 0.00000136 | 0.00022287 |
| *MIR5689HG* | 1.996515 | -1.08481 | 0.00421539 | 0.04966290 |
| *AL451062.1* | 6.100946 | 1.442405 | 0.00003690 | 0.00241468 |
| *KRT16P4* | 8.851965 | -1.02682 | 0.00376430 | 0.04625196 |
| *AL109914.1* | 5.364018 | 1.11972 | 0.00124846 | 0.02366924 |
| *AL078590.1* | 2.372049 | 2.371758 | 0.00014736 | 0.00609189 |
| *TRBV10-2* | 4.435311 | -1.00178 | 0.00199887 | 0.03152519 |
| *AC118754.1* | 1.081524 | -1.02144 | 0.00233756 | 0.03502551 |
| *LINC01435* | 0.864197 | -2.10113 | 0.00010005 | 0.00474650 |
| *ANKRD66* | 32.91778 | -1.69643 | 0.00002740 | 0.00197419 |
| *RPL10P6* | 65.65413 | 1.395059 | 0.00000050 | 0.00010270 |
| *KRT16P5* | 9.189974 | -1.61676 | 0.00003790 | 0.00247152 |
| *TPRG1-AS2* | 1.621556 | 1.260599 | 0.00111207 | 0.02191367 |
| *AC092569.1* | 6.237335 | -1.04177 | 0.00224355 | 0.03404727 |
| *AP000688.1* | 36.19057 | 1.319997 | 0.00000000 | 0.00000303 |
| *PHBP4* | 3.512746 | 1.12223 | 0.00229044 | 0.03455042 |
| *LINC02525* | 6.199747 | -2.70743 | 0.00004320 | 0.00272612 |
| *AOAH-IT1* | 1.856445 | 1.406594 | 0.00040680 | 0.01208221 |
| *AC005722.1* | 1.586435 | 1.646759 | 0.00068893 | 0.01664696 |
| *UQCRHP1* | 1.062827 | -1.09906 | 0.00090414 | 0.01954488 |
| *C2orf27AP3* | 3.29541 | 1.273915 | 0.00163670 | 0.02790516 |
| *AC073347.1* | 11.95464 | 1.997638 | 0.00073468 | 0.01736889 |
| *LINC00326* | 5.489576 | -1.20923 | 0.00288468 | 0.03960993 |
| *LINC01508* | 121.9701 | 1.506599 | 0.00000092 | 0.00016579 |
| *TRBV26OR9-2* | 2.346817 | 1.182647 | 0.00354945 | 0.04481048 |
| *AC007099.1* | 1.280851 | -1.32098 | 0.00212760 | 0.03280881 |
| *APCDD1L-DT* | 48.80081 | -1.27414 | 0.00007910 | 0.00405170 |
| *AC023481.1* | 1.563844 | 1.103466 | 0.00206133 | 0.03213488 |
| *AC108868.1* | 2.448654 | -1.76552 | 0.00004400 | 0.00275818 |
| *ANKRD44-AS1* | 27.40029 | -1.58227 | 0.00000016 | 0.00004270 |
| *SLC47A1P2* | 16.38905 | 1.165675 | 0.00014500 | 0.00603623 |
| *KRT17P6* | 33.24852 | -1.0598 | 0.00013121 | 0.00564518 |
| *AC112907.1* | 2.162497 | 1.099373 | 0.00271817 | 0.03824486 |
| *TSPAN19* | 62.29765 | -1.56469 | 0.00008610 | 0.00430762 |
| *AL512380.1* | 1.461334 | -2.87071 | 0.00003140 | 0.00215953 |
| *DNAJB6P3* | 2.43262 | -1.22953 | 0.00152536 | 0.02676884 |
| *LINC01819* | 6.199312 | -1.33738 | 0.00002160 | 0.00165526 |
| *AL356361.2* | 1.494572 | 1.092134 | 0.00383874 | 0.04673668 |
| *AC108868.2* | 1.268411 | -2.66788 | 0.00094037 | 0.01991271 |
| *AC073488.1* | 18.88997 | 3.135798 | 0.00000000 | 0.00000071 |
| *LINC02043* | 20.31637 | 1.034811 | 0.00017267 | 0.00685718 |
| *ELN-AS1* | 26.77839 | -1.04708 | 0.00002170 | 0.00165797 |
| *LARS2-AS1* | 4.092999 | 1.115297 | 0.00059781 | 0.01520080 |
| *VDAC1P13* | 0.897786 | 1.211063 | 0.00315950 | 0.04200645 |
| *AC021876.1* | 2.682565 | 1.000654 | 0.00418061 | 0.04945290 |
| *ERVH48-1* | 9.452275 | -1.09808 | 0.00010678 | 0.00497231 |
| *AL136307.1* | 4.512849 | 1.575558 | 0.00001400 | 0.00121945 |
| *AC099541.1* | 1.023149 | 1.28439 | 0.00200399 | 0.03156376 |
| *LINC00028* | 1.707479 | -1.91915 | 0.00000021 | 0.00005380 |
| *RPL17P11* | 4.262455 | 1.057425 | 0.00040408 | 0.01202158 |
| *AL035414.1* | 28.79134 | 1.546432 | 0.00028489 | 0.00948029 |
| *LINC01077* | 1.427676 | 1.425029 | 0.00326166 | 0.04263394 |
| *AC093326.1* | 6.104213 | 1.746065 | 0.00142538 | 0.02578238 |
| *AC012506.3* | 1.044511 | 1.677273 | 0.00418077 | 0.04945290 |
| *NRAD1* | 11.25933 | -1.21512 | 0.00048508 | 0.01348514 |
| *LINC01765* | 6.883494 | -1.82645 | 0.00004520 | 0.00278407 |
| *RPL10P9* | 309.2516 | 1.383028 | 0.00000005 | 0.00001760 |
| *LINC01425* | 1.075685 | 2.432427 | 0.00272583 | 0.03829160 |
| *AL138878.2* | 7.424726 | 1.493966 | 0.00000879 | 0.00085557 |
| *LINC01830* | 2.495883 | 4.027629 | 0.00001000 | 0.00094728 |
| *ELK2BP* | 1.979509 | 2.031328 | 0.00306077 | 0.04131218 |
| *AL139042.1* | 3.811374 | 2.188541 | 0.00176023 | 0.02930454 |
| *AL161449.1* | 2.1916 | 1.249276 | 0.00321733 | 0.04236217 |
| *AL355601.1* | 9.213379 | -1.18008 | 0.00023670 | 0.00842475 |
| *LINC01287* | 33.35674 | 2.964802 | 0.00000000 | 0.00000150 |
| *LINC00458* | 1.465214 | 2.573701 | 0.00034269 | 0.01069748 |
| *AL035258.1* | 61.04282 | 1.260563 | 0.00151576 | 0.02670631 |
| *SOX9-AS1* | 50.46283 | -1.53636 | 0.00000001 | 0.00000486 |
| *Z82185.1* | 1.187109 | 1.774724 | 0.00309315 | 0.04149987 |
| *AL512622.1* | 3.512453 | 1.377104 | 0.00004420 | 0.00275818 |
| *AC073842.1* | 2.20918 | 1.073086 | 0.00117531 | 0.02274926 |
| *LINC00330* | 3.261701 | -1.7048 | 0.00009670 | 0.00467948 |
| *FAM237A* | 7.334838 | 1.911274 | 0.00000652 | 0.00070020 |
| *MTFR2P2* | 5.829309 | 1.380404 | 0.00170184 | 0.02865641 |
| *ANKRD34C* | 1.95906 | -1.2702 | 0.00123013 | 0.02349384 |
| *TSPEAR-AS1* | 94.67793 | 1.145984 | 0.00024269 | 0.00857772 |
| *LINC01564* | 120.4702 | 1.186692 | 0.00001190 | 0.00106681 |
| *THRAP3P3* | 1.724796 | 1.757681 | 0.00041932 | 0.01229131 |
| *AC004448.2* | 5.507733 | 1.465346 | 0.00007800 | 0.00401786 |
| *LINC00545* | 1.71351 | -1.65214 | 0.00002400 | 0.00177839 |
| *AL161618.1* | 5.871013 | -1.46193 | 0.00079857 | 0.01819781 |
| *KDM4A-AS1* | 173.1775 | 1.117129 | 0.00000000 | 0.00000182 |
| *RPL26P30* | 32.53472 | -1.24631 | 0.00000002 | 0.00000787 |
| *AC019197.1* | 110.3344 | 1.085535 | 0.00250773 | 0.03635481 |
| *AC019117.2* | 29.17919 | -1.78097 | 0.00000001 | 0.00000385 |
| *TRHDE-AS1* | 15.81202 | -1.56662 | 0.00077266 | 0.01786549 |
| *SCAT8* | 260.0236 | 2.465209 | 0.00000000 | 0.00000000 |
| *AC133473.1* | 2.155601 | 1.165733 | 0.00002420 | 0.00179291 |
| *RPS27AP7* | 1.526231 | 1.109468 | 0.00277753 | 0.03872835 |
| *LINC01010* | 150.4386 | 1.366073 | 0.00000780 | 0.00079527 |
| *LINC00851* | 1.650588 | 2.125866 | 0.00026667 | 0.00907359 |
| *AL080313.2* | 1.524175 | 1.524936 | 0.00096056 | 0.02016678 |
| *PRSS56* | 13.23264 | -1.59682 | 0.00330834 | 0.04294343 |
| *AL512635.1* | 1.590929 | 1.639819 | 0.00082032 | 0.01847893 |
| *BHMG1* | 2.352423 | 1.220559 | 0.00009330 | 0.00457374 |
| *PYY2* | 126.0976 | 1.163754 | 0.00050931 | 0.01384705 |
| *GSTA7P* | 12.9267 | 1.480459 | 0.00030763 | 0.00994541 |
| *LINC01143* | 7.319524 | 1.288471 | 0.00097377 | 0.02029911 |
| *LINC01370* | 11.41482 | 2.719317 | 0.00015505 | 0.00632088 |
| *AL035425.1* | 66.3328 | 1.646427 | 0.00004730 | 0.00285859 |
| *NFIA-AS2* | 6.78054 | -1.10843 | 0.00001970 | 0.00154962 |
| *AL606970.4* | 11.60688 | 1.388679 | 0.00312940 | 0.04183259 |
| *AL627309.1* | 2.277136 | 1.515158 | 0.00026725 | 0.00907359 |
| *AC078993.1* | 11.92624 | 1.524984 | 0.00000071 | 0.00013366 |
| *OR2W3* | 5.662195 | 1.761683 | 0.00002140 | 0.00164805 |
| *U8* | 25.0006 | 1.568704 | 0.00006750 | 0.00359137 |
| *RNU2-11P* | 4.882798 | 1.006505 | 0.00006390 | 0.00349841 |
| *ATP6V1B1-AS1* | 8.099083 | 1.478113 | 0.00000016 | 0.00004300 |
| *LINC02004* | 5.869278 | -1.16241 | 0.00053070 | 0.01417559 |
| *PCDHGC5* | 36.43031 | -1.25124 | 0.00000081 | 0.00014608 |
| *IQCJ-SCHIP1-AS1* | 2.294864 | 1.019779 | 0.00131200 | 0.02436630 |
| *AC106712.1* | 6.257658 | -1.50265 | 0.00002160 | 0.00165526 |
| *SLC25A24P1* | 11.34515 | 1.519087 | 0.00218798 | 0.03347696 |
| *AL158847.1* | 2.524284 | 1.675976 | 0.00000263 | 0.00037159 |
| *ARHGDIG* | 20.06493 | 1.314201 | 0.00004470 | 0.00277495 |
| *AC121764.1* | 10.47829 | 2.883102 | 0.00000171 | 0.00026902 |
| *LINC01206* | 1525.586 | 3.419826 | 0.00000000 | 0.00000000 |
| *IGKV1D-17* | 186.9564 | -1.25293 | 0.00105559 | 0.02129730 |
| *SOX2-OT* | 1538.041 | 1.793601 | 0.00000006 | 0.00001940 |
| *AC068756.1* | 5.473761 | 2.102287 | 0.00078668 | 0.01805492 |
| *STRC* | 14.11268 | 1.08752 | 0.00001570 | 0.00130382 |
| *AADACL2-AS1* | 23.28996 | 1.021638 | 0.00204378 | 0.03192015 |
| *AL161757.1* | 2.663641 | 1.277807 | 0.00318646 | 0.04221222 |
| *LINC00870* | 3.800697 | 1.147343 | 0.00106780 | 0.02143003 |
| *TUSC7* | 6.331882 | 2.621058 | 0.00144309 | 0.02594621 |
| *IGKV2-30* | 274.0463 | -1.09243 | 0.00020946 | 0.00778475 |
| *MNX1-AS1* | 88.77378 | 1.144443 | 0.00037784 | 0.01153228 |
| *WFDC6* | 5.579093 | -1.6465 | 0.00266916 | 0.03782448 |
| *CFAP57* | 143.7103 | -1.22533 | 0.00000107 | 0.00018452 |
| *CACNA2D3-AS1* | 3.336157 | 1.309967 | 0.00107904 | 0.02153391 |
| *TUBA4B* | 57.77966 | -1.09386 | 0.00022318 | 0.00809046 |
| *GSTA1* | 5490.782 | 1.873289 | 0.00000062 | 0.00012057 |
| *AC108676.1* | 201.8513 | 1.159161 | 0.00182595 | 0.02985030 |
| *AL109761.1* | 7.755494 | 1.014757 | 0.00037738 | 0.01152824 |
| *DRAIC* | 27.46324 | 1.349471 | 0.00000123 | 0.00020868 |
| *AC106793.1* | 3.263185 | 1.11394 | 0.00093358 | 0.01989964 |
| *LINC01513* | 4.288108 | -1.07932 | 0.00137913 | 0.02521023 |
| *LINC00920* | 69.87144 | -1.02068 | 0.00000148 | 0.00023785 |
| *SNCA-AS1* | 1.839324 | 1.438165 | 0.00159461 | 0.02755980 |
| *AC114812.3* | 3.870463 | 1.056286 | 0.00167941 | 0.02842769 |
| *GLDCP1* | 2.558338 | 1.404161 | 0.00244809 | 0.03597730 |
| *C4orf54* | 28.74053 | 1.233939 | 0.00032470 | 0.01031115 |
| *AC037441.1* | 4.442755 | -1.26225 | 0.00120927 | 0.02321595 |
| *LINC02265* | 6.153459 | -1.11346 | 0.00002060 | 0.00160547 |
| *LINC00939* | 20.88047 | 1.718338 | 0.00000781 | 0.00079527 |
| *LINC01088* | 61.47759 | -1.3126 | 0.00000399 | 0.00050445 |
| *AC010468.1* | 0.831504 | 1.353167 | 0.00192192 | 0.03079236 |
| *AL033397.2* | 46.22414 | 1.363545 | 0.00000001 | 0.00000578 |
| *LINC02071* | 1.515109 | 1.473683 | 0.00082807 | 0.01859431 |
| *LINC02147* | 2.561029 | -1.36661 | 0.00002530 | 0.00184910 |
| *LINC00536* | 8.071976 | -1.13298 | 0.00174747 | 0.02918838 |
| *CCT7P2* | 1.252563 | 1.66967 | 0.00341331 | 0.04374190 |
| *SLC7A11-AS1* | 38.05654 | 1.239582 | 0.00029681 | 0.00968631 |
| *AC096719.1* | 8.629302 | 1.420785 | 0.00002960 | 0.00206171 |
| *AC093844.1* | 13.01567 | 1.329972 | 0.00188890 | 0.03042907 |
| *LINC00989* | 3.099822 | -1.07014 | 0.00063663 | 0.01585708 |
| *TUNAR* | 4.33691 | 2.383563 | 0.00189545 | 0.03050396 |
| *AACSP1* | 28.40795 | -1.49455 | 0.00008350 | 0.00420430 |
| *KNOP1P5* | 9.076453 | 1.545407 | 0.00366500 | 0.04565940 |
| *AC109454.3* | 34.84073 | 1.244326 | 0.00411926 | 0.04893423 |
| *SNHG18* | 584.3037 | -1.14423 | 0.00000060 | 0.00011822 |
| *LINC02111* | 3.490245 | 1.591241 | 0.00049734 | 0.01370081 |
| *EPHA5-AS1* | 6.113392 | 1.854534 | 0.00080417 | 0.01825481 |
| *LINC02208* | 6.123696 | 1.148088 | 0.00176238 | 0.02932652 |
| *AC105460.1* | 139.174 | 1.685771 | 0.00368049 | 0.04575559 |
| *LINC02382* | 6.310718 | 1.718207 | 0.00055038 | 0.01449528 |
| *LMNB1-DT* | 1.406738 | 1.02337 | 0.00193123 | 0.03085779 |
| *AC025539.1* | 17.08578 | -1.05168 | 0.00050480 | 0.01378804 |
| *AC105460.2* | 9.318838 | 1.560151 | 0.00019447 | 0.00739854 |
| *AC055854.1* | 7.378938 | -1.23509 | 0.00052201 | 0.01403029 |
| *AC100801.1* | 6.300983 | -1.90592 | 0.00023797 | 0.00845323 |
| *IGLV2-5* | 23.16455 | -1.02001 | 0.00077007 | 0.01783733 |
| *PCDHGB6* | 97.26372 | -1.21656 | 0.00000000 | 0.00000004 |
| *AC008514.1* | 13.57097 | -1.75029 | 0.00000038 | 0.00008550 |
| *AC044893.1* | 10.40088 | -2.71901 | 0.00001340 | 0.00118543 |
| *KBTBD11-OT1* | 3.017853 | 1.216942 | 0.00052057 | 0.01401534 |
| *SIRLNT* | 15.89229 | 3.902435 | 0.00000014 | 0.00004030 |
| *LINC01151* | 2.910964 | -1.69925 | 0.00005390 | 0.00312000 |
| *LINC01419* | 17.90272 | 2.150906 | 0.00079848 | 0.01819781 |
| *LINC02055* | 21.02035 | 1.651708 | 0.00187785 | 0.03037565 |
| *CASC19* | 83.08921 | 1.283621 | 0.00022744 | 0.00820463 |
| *AC107953.2* | 2.025163 | 1.72096 | 0.00030812 | 0.00994541 |
| *AC124067.4* | 4.005621 | -1.2815 | 0.00096094 | 0.02016678 |
| *GLYATL1P4* | 1.81874 | -1.56548 | 0.00244151 | 0.03594032 |
| *AL358944.1* | 2.368132 | -1.11029 | 0.00210440 | 0.03259325 |
| *AC036111.1* | 1.431899 | 3.048666 | 0.00021453 | 0.00789812 |
| *AC105219.1* | 7.957518 | 1.200748 | 0.00002180 | 0.00166075 |
| *AP003119.1* | 27.0274 | 1.235777 | 0.00393038 | 0.04744349 |
| *AC009646.2* | 2.566488 | 1.312039 | 0.00078144 | 0.01798097 |
| *LINC02551* | 17.50171 | -1.0949 | 0.00078016 | 0.01796360 |
| *AF131216.3* | 42.5103 | 1.011822 | 0.00376327 | 0.04625196 |
| *AC093496.1* | 5.420161 | 2.348974 | 0.00001170 | 0.00105886 |
| *AF186192.2* | 19.20542 | 1.091158 | 0.00052438 | 0.01406202 |
| *GLYATL1P1* | 3.118155 | -1.19689 | 0.00012424 | 0.00545291 |
| *ENPP7P8* | 2.729045 | 1.700835 | 0.00004040 | 0.00258983 |
| *AC108136.1* | 15.71267 | -1.23235 | 0.00004670 | 0.00284759 |
| *SMILR* | 20.522 | -1.37906 | 0.00000120 | 0.00020424 |
| *LINC02764* | 1.881366 | 1.354801 | 0.00251061 | 0.03638169 |
| *LINC02389* | 6.264768 | 1.02021 | 0.00084753 | 0.01872187 |
| *AC022509.1* | 13.00921 | -1.55309 | 0.00002970 | 0.00206171 |
| *AP000439.2* | 5.665762 | 1.835133 | 0.00217546 | 0.03333844 |
| *AC125616.1* | 6.311297 | 1.395938 | 0.00029292 | 0.00962159 |
| *AC022509.2* | 26.8239 | -1.2191 | 0.00000046 | 0.00009580 |
| *AC005832.1* | 1.728718 | 1.122647 | 0.00089454 | 0.01945405 |
| *ASIC5* | 4.67222 | 1.090802 | 0.00059129 | 0.01511256 |
| *AP006333.2* | 1.028068 | 1.330604 | 0.00360822 | 0.04528434 |
| *SLC5A8* | 119.4588 | -1.1672 | 0.00115049 | 0.02240375 |
| *AP000851.1* | 13.66813 | -1.92386 | 0.00000000 | 0.00000271 |
| *AC084375.1* | 13.38726 | -1.32492 | 0.00028990 | 0.00957602 |
| *C11orf97* | 11.23998 | -1.36764 | 0.00314136 | 0.04191320 |
| *LINC02874* | 5.18221 | 1.578253 | 0.00000207 | 0.00031264 |
| *DYNLL1P4* | 6.672714 | 1.418615 | 0.00000066 | 0.00012738 |
| *LINC02388* | 4.423877 | -1.95285 | 0.00009590 | 0.00466219 |
| *AC025575.1* | 2.904696 | 1.041942 | 0.00107267 | 0.02145573 |
| *AC009320.1* | 1.185563 | 1.008869 | 0.00278103 | 0.03872835 |
| *AC025154.2* | 25.50503 | -1.68764 | 0.00000511 | 0.00058887 |
| *AC004024.1* | 9.33187 | 1.122626 | 0.00010564 | 0.00494512 |
| *AC125603.1* | 1.344307 | 2.394946 | 0.00105996 | 0.02135847 |
| *AC089983.1* | 44.69122 | 2.34854 | 0.00000000 | 0.00000003 |
| *AC125603.2* | 4.664284 | 2.637408 | 0.00002850 | 0.00202563 |
| *AC078864.1* | 4.810475 | -2.41831 | 0.00003410 | 0.00228176 |
| *AC078820.1* | 8.337753 | -1.16637 | 0.00128634 | 0.02410233 |
| *AC012085.2* | 5.027188 | 1.070385 | 0.00052388 | 0.01405917 |
| *AL160191.1* | 1.273888 | 1.613997 | 0.00330848 | 0.04294343 |
| *AC026495.1* | 5.063226 | 1.190538 | 0.00186962 | 0.03031190 |
| *LINC01269* | 45.13892 | -1.069 | 0.00042751 | 0.01247725 |
| *AL137786.1* | 15.8113 | 1.00312 | 0.00202880 | 0.03177043 |
| *LINC01193* | 2.950661 | 2.54587 | 0.00101165 | 0.02080034 |
| *AL357093.2* | 38.78512 | -1.73792 | 0.00000533 | 0.00060405 |
| *LINC01579* | 78.75872 | 1.270723 | 0.00004700 | 0.00284759 |
| *AC004828.1* | 1.452457 | 1.518008 | 0.00029107 | 0.00960095 |
| *AC007040.2* | 14.71966 | 1.203711 | 0.00000035 | 0.00008140 |
| *TMEM179* | 104.7631 | 1.855156 | 0.00000849 | 0.00083383 |
| *LINC00639* | 38.57321 | -1.77029 | 0.00000000 | 0.00000003 |
| *AC046168.1* | 8.96987 | -1.67333 | 0.00000412 | 0.00051306 |
| *AC012050.1* | 1.15779 | 1.831947 | 0.00044191 | 0.01270863 |
| *LINC02284* | 2.207629 | 1.164593 | 0.00062374 | 0.01565540 |
| *AC106738.2* | 1.232364 | -1.40593 | 0.00066932 | 0.01638853 |
| *AC090907.2* | 12.59385 | 1.959635 | 0.00000000 | 0.00000201 |
| *FIGNL2-DT* | 1.54393 | -1.02006 | 0.00175082 | 0.02920758 |
| *AC093802.2* | 5.284777 | 2.741664 | 0.00076411 | 0.01777934 |
| *AC130456.2* | 14.05769 | -1.08496 | 0.00006680 | 0.00357844 |
| *AC007342.1* | 1.086576 | 1.09068 | 0.00175193 | 0.02920758 |
| *AC034111.1* | 1.057199 | 1.408289 | 0.00100859 | 0.02076160 |
| *AGBL1-AS1* | 4.014297 | -1.36734 | 0.00243431 | 0.03593908 |
| *TPSP2* | 8.941961 | 1.334221 | 0.00189895 | 0.03052143 |
| *AL360014.1* | 7.126252 | 1.345628 | 0.00023751 | 0.00844522 |
| *AC009065.3* | 0.875161 | 1.053037 | 0.00300946 | 0.04081032 |
| *AL355607.2* | 7.944385 | -1.02001 | 0.00369614 | 0.04586948 |
| *AL365181.1* | 0.960391 | 1.311079 | 0.00372943 | 0.04602006 |
| *INSYN1-AS1* | 24.79211 | 1.396343 | 0.00028711 | 0.00952817 |
| *AC116348.1* | 4.121651 | -1.09117 | 0.00222107 | 0.03380773 |
| *AC126696.3* | 4.82073 | 1.153992 | 0.00006190 | 0.00343813 |
| *AC004158.1* | 1.673117 | -1.16985 | 0.00364811 | 0.04559362 |
| *AC007823.1* | 34.294 | 1.090845 | 0.00000000 | 0.00000001 |
| *AC093520.1* | 13.70122 | 1.080561 | 0.00000286 | 0.00039474 |
| *CES1P2* | 6.359213 | 1.573088 | 0.00025761 | 0.00889157 |
| *CEROX1* | 62.94817 | -1.21936 | 0.00000007 | 0.00002240 |
| *LINC02555* | 30.81806 | -1.15974 | 0.00007990 | 0.00406759 |
| *WFDC21P* | 1503.354 | -1.17767 | 0.00007840 | 0.00403243 |
| *LINC02178* | 14.17184 | -1.2599 | 0.00180093 | 0.02969206 |
| *AC007159.1* | 1.317269 | -3.3211 | 0.00053808 | 0.01425636 |
| *LINC01989* | 3.814929 | 2.239143 | 0.00000279 | 0.00038871 |
| *LINC02188* | 61.60035 | -1.62009 | 0.00001040 | 0.00097553 |
| *ANKRD26P1* | 2.303205 | -1.50195 | 0.00085339 | 0.01877364 |
| *AC116348.2* | 8.714841 | -1.19194 | 0.00273164 | 0.03834278 |
| *AP000439.3* | 0.941582 | -3.27086 | 0.00328698 | 0.04285325 |
| *AL035425.3* | 178.0821 | 2.210273 | 0.00000044 | 0.00009400 |
| *AC134682.1* | 20.50843 | 1.041324 | 0.00000720 | 0.00075694 |
| *AC010531.4* | 1.595216 | 1.326455 | 0.00046988 | 0.01324350 |
| *AL133383.1* | 2.702065 | -1.12225 | 0.00196558 | 0.03115305 |
| *AC244090.2* | 7.560759 | 1.027903 | 0.00013248 | 0.00566861 |
| *MIA* | 45.78766 | -2.557 | 0.00000000 | 0.00000001 |
| *LINC01996* | 4.273227 | -1.14531 | 0.00197135 | 0.03121632 |
| *AL079343.1* | 1.774596 | 1.601208 | 0.00003380 | 0.00227358 |
| *AC104564.1* | 2.233618 | 1.00037 | 0.00013063 | 0.00563745 |
| *AKR1C8P* | 7.760306 | 1.172753 | 0.00017474 | 0.00692382 |
| *AC104564.2* | 4.022154 | 1.014479 | 0.00015068 | 0.00619491 |
| *AC145207.8* | 61.42746 | 1.283006 | 0.00003270 | 0.00221443 |
| *PPY2P* | 9.25096 | 2.32732 | 0.00025459 | 0.00883038 |
| *AC104984.2* | 0.913115 | 1.140352 | 0.00106995 | 0.02143003 |
| *LINC01029* | 1.580582 | 2.39711 | 0.00116297 | 0.02257220 |
| *LINC01387* | 3.217693 | 1.61631 | 0.00181474 | 0.02976341 |
| *ESRG* | 210.078 | 2.644753 | 0.00000318 | 0.00042913 |
| *AC009779.1* | 1.781923 | 2.540452 | 0.00022825 | 0.00821496 |
| *AP001011.1* | 5.583438 | 1.67308 | 0.00045722 | 0.01302646 |
| *AC015813.2* | 0.907743 | 1.277283 | 0.00064553 | 0.01602224 |
| *AP002478.1* | 45.37364 | 1.119629 | 0.00090138 | 0.01950514 |
| *AC061975.6* | 30.14881 | 2.544279 | 0.00000253 | 0.00036338 |
| *AC011511.2* | 2.495389 | -1.14225 | 0.00220448 | 0.03362852 |
| *TCF4-AS1* | 18.30114 | 1.344704 | 0.00013418 | 0.00572697 |
| *AC005336.1* | 868.344 | 1.325178 | 0.00114637 | 0.02234829 |
| *AC008738.2* | 5.524622 | 1.167862 | 0.00000263 | 0.00037159 |
| *LCN6* | 3.328212 | -1.03304 | 0.00148110 | 0.02634830 |
| *AC008649.1* | 36.40034 | 1.211358 | 0.00037210 | 0.01138980 |
| *RPL23AP77* | 4.762677 | -1.30361 | 0.00004200 | 0.00266207 |
| *AC008759.1* | 1.194997 | -1.4954 | 0.00013449 | 0.00572697 |
| *AC011511.5* | 30.75925 | -1.01811 | 0.00000072 | 0.00013366 |
| *CCDC177* | 75.04538 | 1.779185 | 0.00000008 | 0.00002410 |
| *AC010605.1* | 3.731987 | 1.08498 | 0.00001080 | 0.00101112 |
| *MIA-RAB4B* | 0.976521 | -1.5845 | 0.00281748 | 0.03897058 |
| *AC063977.6* | 5.62964 | 1.077693 | 0.00027947 | 0.00935790 |
| *AC011503.1* | 9.350457 | 1.126247 | 0.00101556 | 0.02086852 |
| *AC092071.1* | 5.191256 | -1.08776 | 0.00223664 | 0.03397161 |
| *AL021328.1* | 14.98836 | -1.47117 | 0.00000640 | 0.00069352 |
| *BNIP3P9* | 1.241909 | 1.518219 | 0.00319682 | 0.04227917 |
| *GAS2L2* | 41.36955 | -1.12322 | 0.00050354 | 0.01378545 |
| *SREK1IP1P2* | 3.861316 | 2.100741 | 0.00002340 | 0.00174972 |
| *SRXN1* | 963.1728 | 1.225774 | 0.00000160 | 0.00025495 |
| *AC019080.3* | 18.81397 | 1.440845 | 0.00000134 | 0.00022107 |
| *AC016747.2* | 6.865547 | 1.043342 | 0.00018597 | 0.00718338 |
| *AL365181.2* | 220.263 | 1.282511 | 0.00103981 | 0.02118219 |
| *U4* | 5.453865 | 1.056011 | 0.00000758 | 0.00078589 |
| *AL365181.4* | 4.59408 | 1.488181 | 0.00156943 | 0.02728560 |
| *LINC02012* | 76.77584 | 1.076073 | 0.00000811 | 0.00081708 |
| *AL355312.3* | 32.95626 | -1.55147 | 0.00000000 | 0.00000220 |
| *AL359962.2* | 13.03959 | 1.083927 | 0.00002560 | 0.00186705 |
| *AL391069.2* | 1.240218 | 1.062552 | 0.00379672 | 0.04645509 |
| *AC007204.1* | 1.189075 | 1.263679 | 0.00117934 | 0.02281473 |
| *AL513485.1* | 7.662067 | 1.101349 | 0.00385060 | 0.04686482 |
| *ADRA2B* | 363.0481 | 1.860669 | 0.00000000 | 0.00000012 |
| *AL049757.1* | 7.766659 | 1.14698 | 0.00006800 | 0.00360304 |
| *LINC01297* | 8.541792 | 2.200163 | 0.00022918 | 0.00823182 |
| *AC018445.1* | 1.255337 | -1.92087 | 0.00153966 | 0.02693947 |
| *AL080312.2* | 1.592633 | 1.074178 | 0.00178497 | 0.02951038 |
| *HNF1B* | 109.3124 | -1.46845 | 0.00000019 | 0.00005010 |
| *AC007998.4* | 6.256256 | 1.408605 | 0.00003170 | 0.00216216 |
| *AL590491.2* | 2.26422 | -1.71278 | 0.00143126 | 0.02580937 |
| *FAM25C* | 1.73595 | -1.57834 | 0.00186869 | 0.03031190 |
| *AL136221.1* | 6.130126 | 1.094226 | 0.00009210 | 0.00453259 |
| *YWHAEP7* | 3.958017 | 1.066708 | 0.00393170 | 0.04744349 |
| *AC002401.4* | 14.99304 | -1.34988 | 0.00003650 | 0.00239974 |
| *AL133325.3* | 22.73328 | 1.348444 | 0.00107211 | 0.02145573 |
| *AC023490.4* | 1.369136 | -1.84045 | 0.00005410 | 0.00312261 |
| *AL591424.3* | 1.010952 | 1.820887 | 0.00299012 | 0.04062775 |
| *AC133540.1* | 4.228019 | 1.745423 | 0.00000040 | 0.00008690 |
| *AC020765.4* | 1.343912 | 1.092683 | 0.00235458 | 0.03517169 |
| *AC097460.2* | 1.007882 | 1.508427 | 0.00011359 | 0.00514716 |
| *AC092135.1* | 2.25158 | 1.138593 | 0.00278493 | 0.03874117 |
| *LINC01666* | 6.564688 | 1.439133 | 0.00108248 | 0.02156655 |
| *Z82217.1* | 13.44695 | 1.005541 | 0.00000015 | 0.00004170 |
| *AC015959.1* | 2.668992 | 1.138138 | 0.00119596 | 0.02302289 |
| *AC018470.1* | 12.17002 | 1.17018 | 0.00175976 | 0.02930454 |
| *KCNIP4-IT1* | 0.917167 | 1.675168 | 0.00058596 | 0.01505086 |
| *PCAT5* | 3.53063 | 1.388452 | 0.00395647 | 0.04766104 |
| *JAKMIP2-AS1* | 10.41485 | -1.14726 | 0.00217089 | 0.03330204 |
| *FOXCUT* | 7.166873 | -1.23442 | 0.00001800 | 0.00144566 |
| *PTENP1-AS* | 8.440301 | 1.310164 | 0.00049555 | 0.01366191 |
