# Supplementary material for: Construction and validation of a novel prognostic model for lung squamous cell cancer based on N6-methyladenosine-related genes
Source: World J Surg Oncol. 2022 Feb 27;20:59. doi: 10.1186/s12957-022-02509-1 (PMC8883700; doi:10.1186/s12957-022-02509-1)
Supplement: Supplementary file 2 — Additional file 2 : Supplement Table 2. Results of univariate Cox analysis in TCGA LUSC. [file 12957_2022_2509_MOESM2_ESM.docx]

| Supplement table 2. Results of univariate Cox analysis in TCGA LUSC | | | | |
| --- | --- | --- | --- | --- |
| Gene symbol | HR | ci_lower | ci_upper | p_value |
| SCIN | 0.873728432 | 0.753376543 | 1.013306533 | 0.068927505 |
| TKTL1 | 1.082577791 | 0.990349129 | 1.18339547 | 0.095993252 |
| CYP24A1 | 1.138768228 | 1.024432936 | 1.265864295 | 0.01687279 |
| KRT23 | 1.129918781 | 1.030385016 | 1.23906737 | 0.009417396 |
| GRM4 | 1.114292046 | 0.993463058 | 1.249816744 | 0.066228659 |
| EREG | 1.124536909 | 0.996628557 | 1.268861152 | 0.06381165 |
| TMEM255A | 0.842410306 | 0.710064383 | 0.99942363 | 0.044263209 |
| H19 | 1.124315794 | 1.021969118 | 1.236912136 | 0.019416114 |
| FAM71F1 | 0.900239631 | 0.806187724 | 1.005263873 | 0.056347146 |
| DPEP3 | 1.106536791 | 1.015636913 | 1.205572241 | 0.035425664 |
| ADCY10 | 0.88427231 | 0.789472208 | 0.990456042 | 0.031493254 |
| NXF3 | 1.133140207 | 0.989614619 | 1.297481569 | 0.078809029 |
| PLPPR1 | 0.885883095 | 0.776174242 | 1.011098817 | 0.073021109 |
| CBR1 | 0.816917893 | 0.690921927 | 0.965890382 | 0.018372667 |
| ACTC1 | 0.855294079 | 0.722896976 | 1.011939441 | 0.065053675 |
| PTGER1 | 1.270039564 | 1.066228088 | 1.512809981 | 0.007528433 |
| TRIM58 | 1.249605956 | 1.106811442 | 1.410823007 | 0.000589338 |
| MT1E | 1.226037862 | 1.026532476 | 1.464316885 | 0.023835097 |
| CLDN20 | 0.838332747 | 0.726038007 | 0.967995873 | 0.012575853 |
| MYEOV | 1.111513416 | 0.99233082 | 1.245010283 | 0.071551607 |
| CNTNAP2 | 0.898761784 | 0.819411289 | 0.985796456 | 0.02899879 |
| SLC35G1 | 0.823711224 | 0.682877056 | 0.993590536 | 0.039251902 |
| ALOX15B | 1.171618333 | 0.973175406 | 1.410526313 | 0.097751007 |
| WDR97 | 1.304073718 | 1.116875501 | 1.522648013 | 0.00094736 |
| MGAT4C | 0.876046525 | 0.765208943 | 1.002938507 | 0.048742815 |
| RNASE10 | 1.135960073 | 0.999916661 | 1.290512837 | 0.05474754 |
| POU3F2 | 0.897548353 | 0.802059442 | 1.004405663 | 0.04587345 |
| MUC6 | 1.131994672 | 0.988035417 | 1.296929153 | 0.085780549 |
| WASIR1 | 1.203471687 | 0.984708863 | 1.470834839 | 0.085982569 |
| HTR3E | 0.808300365 | 0.644334547 | 1.013991075 | 0.057126956 |
| CLCN1 | 1.146503555 | 0.992280492 | 1.324696407 | 0.065341039 |
| RORB | 0.827636451 | 0.704864837 | 0.97179212 | 0.014725395 |
| AC109583.1 | 1.106866511 | 1.018751392 | 1.202602993 | 0.018409329 |
| GSTA8P | 0.917271808 | 0.836582746 | 1.005743393 | 0.058965408 |
| AC114489.2 | 0.871747568 | 0.748199949 | 1.01569617 | 0.083589496 |
| AC012363.1 | 1.231575725 | 1.06433452 | 1.425095905 | 0.009562417 |
| RORB-AS1 | 0.710168757 | 0.523265671 | 0.963830977 | 0.010248086 |
| LINC02470 | 0.807622617 | 0.629879245 | 1.035522757 | 0.076042276 |
| AC244107.1 | 1.168145079 | 1.018102885 | 1.340299636 | 0.0389046 |
| AL596223.1 | 1.140337938 | 0.989361956 | 1.314352756 | 0.08383756 |
| RPL10P6 | 0.838959748 | 0.697528408 | 1.009067805 | 0.04137853 |
| AP000688.1 | 0.838372266 | 0.693259283 | 1.013860288 | 0.065258176 |
| AL136307.1 | 1.204491866 | 0.993791971 | 1.459863531 | 0.06542176 |
| AC012506.3 | 0.725676252 | 0.48765173 | 1.079881378 | 0.082757822 |
| RPL10P9 | 0.823420864 | 0.670018443 | 1.011945158 | 0.048217449 |
| AL138878.2 | 0.832466714 | 0.685228422 | 1.011342798 | 0.055024827 |
| LINC01287 | 1.186000146 | 1.061124663 | 1.325571249 | 0.006078174 |
| AL035258.1 | 0.900110877 | 0.814419828 | 0.994818107 | 0.04075516 |
| FAM237A | 0.834712673 | 0.704880673 | 0.988458434 | 0.028329177 |
| TRHDE-AS1 | 1.130447131 | 1.015725724 | 1.258125777 | 0.031766109 |
| AL606970.4 | 0.88833519 | 0.771613395 | 1.022713466 | 0.089445783 |
| OR2W3 | 1.314165847 | 1.14779058 | 1.504657647 | 0.000244181 |
| EPHA5-AS1 | 0.84557776 | 0.700365687 | 1.020897742 | 0.060361073 |
| PCDHGB6 | 1.271934041 | 1.013565931 | 1.59616277 | 0.044276044 |
| LINC02055 | 1.114633416 | 1.003198333 | 1.238446687 | 0.053782728 |
| AC105219.1 | 1.273408788 | 1.08905263 | 1.488972982 | 0.003341938 |
| AC009646.2 | 1.193279512 | 0.97815125 | 1.455721693 | 0.092903447 |
| AF131216.3 | 0.904890213 | 0.809894765 | 1.011028015 | 0.080693991 |
| GLYATL1P1 | 0.808373206 | 0.628554849 | 1.039634396 | 0.090003405 |
| AC090907.2 | 1.127387096 | 0.984198592 | 1.291407725 | 0.094883725 |
| AC009065.3 | 1.876697303 | 1.380047693 | 2.552080472 | 0.000135984 |
| AL355607.2 | 1.148253991 | 1.003120705 | 1.314385419 | 0.057886024 |
| LINC02178 | 1.143120335 | 1.004475719 | 1.30090163 | 0.046006842 |
| LINC02188 | 1.134115211 | 1.02454465 | 1.255403865 | 0.01794079 |
| AC010531.4 | 1.27691239 | 1.027351797 | 1.587095342 | 0.042884709 |
| AC244090.2 | 0.845699354 | 0.704975644 | 1.014513627 | 0.063512865 |
| AC061975.6 | 1.112934318 | 0.994863659 | 1.24501763 | 0.075198993 |
| AC008738.2 | 1.223068582 | 0.97819146 | 1.52924741 | 0.074239844 |
| CCDC177 | 0.812866344 | 0.712456593 | 0.927427299 | 0.001683094 |
| BNIP3P9 | 1.308150105 | 0.983718425 | 1.739579797 | 0.085169532 |
| AL136221.1 | 1.187139312 | 0.98594444 | 1.429390632 | 0.077163966 |
| JAKMIP2-AS1 | 0.83161068 | 0.694270207 | 0.996119834 | 0.035999334 |
| FOXCUT | 1.16547735 | 0.984841767 | 1.37924436 | 0.07824588 |

| Supplement table 2. Results of univariate Cox analysis in GEO LUSC | | | | |
| --- | --- | --- | --- | --- |
| Gene symbol | HR | ci_lower | ci_upper | p_value |
| *MT1E* | 35.60406408 | 4.169305391 | 304.0433021 | 0.001377945 |
| *POU3F2* | 79.27085368 | 3.190659999 | 1969.45718 | 0.007941638 |
| *CNTNAP2* | 24.27714132 | 1.831294016 | 32.18377745 | 0.016246097 |
| *EREG* | 0.139762916 | 0.023195402 | 0.842135538 | 0.032502223 |
| *FAM71F1* | 0.090779194 | 0.011161805 | 0.738309106 | 0.024803021 |
| *MYEOV* | 5.874867452 | 1.590659161 | 21.69796549 | 0.011552483 |
| *GRM4* | 0.128157212 | 0.012935101 | 1.269744285 | 0.084814981 |
| *HTR3E* | 0.278740361 | 0.066173211 | 1.174133571 | 0.078882843 |
